# The Evolution of Cognitive Control in Lemurs

**DOI:** 10.1177/09567976221082938

**Published:** 2022-07-25

**Authors:** Francesca De Petrillo, Parvathy Nair, Averill Cantwell, Alexandra G. Rosati

**Affiliations:** 1 Department of Psychology, University of Michigan; 2 School of Psychology, Newcastle University; 3 Biosciences Institute, Newcastle University; 4 Department of Anthropology, University of Michigan

**Keywords:** executive function, comparative cognition, primates, cognitive evolution, open materials, preregistered

## Abstract

Cognitive control, or executive function, is a key feature of human cognition, allowing individuals to plan, acquire new information, or adopt new strategies when the circumstances change. Yet it is unclear which factors promote the evolution of more sophisticated executive-function abilities such as those possessed by humans. Examining cognitive control in nonhuman primates, our closest relatives, can help to identify these evolutionary processes. Here, we developed a novel battery to experimentally measure multiple aspects of cognitive control in primates: temporal discounting, motor inhibition, short-term memory, reversal learning, novelty responses, and persistence. We tested lemur species with targeted, independent variation in both ecological and social features (ruffed lemurs, Coquerel’s sifakas, ring-tailed lemurs, and mongoose lemurs; *N* = 39) and found that ecological rather than social characteristics best predicted patterns of cognitive control across these species. This highlights the importance of integrating cognitive data with species’ natural history to understand the origins of complex cognition.

Cognitive control (also known as *executive function*) refers to a set of top-down processes that include inhibition, updating, and working memory (Diamond, 2013; Friedman & Miyake, 2017). These regulatory cognitive processes enable flexible, goal-directed behaviors and reflect a key distinction between making more reflexive responses and overcoming immediate reactions in order to modulate behavior in service of an overarching goal. These processes are thus benchmark components of intelligent behavior that allow individuals to adjust their actions so they are appropriate in the current context. These cognitive abilities are further thought to be especially elaborated in humans, both because our species appears to show highly flexible behavior compared with other animals (Laland & Seed, 2021) and because cognitive control recruits brain regions such as prefrontal cortex that are evolutionarily labile in primates and expanded in humans specifically (Bush & Allman, 2004; Schoenemann, 2006).

Why does robust cognitive control sometimes emerge across species, such as in humans? There are two major hypotheses for the emergence of intelligent behavior. The *social-intelligence hypothesis* is the dominant view and proposes that social complexity drives cognitive evolution (Byrne & Whiten, 1988; Dunbar, 1998; Moll & Tomasello, 2007; van Schaik & Burkart, 2011). Various social challenges—such as maintaining multiple relationships in a large complex group, outcompeting other individuals, and cooperating or learning from others—have all been proposed as evolutionary drivers of larger brains and enhanced cognition (Dunbar, 1998; Byrne & Whiten, 1988; Moll & Tomasello, 2007; van Schaik & Burkart, 2011). In contrast, the *ecological-intelligence hypothesis* posits that ecological challenges spur cognitive evolution. For example, species that experience environmental fluctuation or rely on foods that have a more heterogenous spatial or temporal distribution may develop more sophisticated cognitive capacities to track resources in the environment (DeCasien et al., 2017; Milton, 1981; Rosati, 2017b; van Woerden et al., 2010). Although the social-intelligence hypothesis has predominated for several decades, there is increasing support for the ecological-intelligence hypothesis, as both larger brains as well as some cognitive features are best predicted by diet (DeCasien et al., 2017; Powell et al., 2017; Rosati, 2017b).

However, prior tests of the social- and ecological-intelligence hypotheses have been limited in several respects. First, a dominant approach has been to use brain size, rather than direct assessments of cognition, as a proxy for cognition (DeCasien et al., 2017; Dunbar, 1998), but broad neuroanatomical measures are only an approximate index for specific cognitive traits (Logan et al., 2018). Direct tests of cognition have heavily focused on individual species, or pairs of species, which limits evolutionary inferences (e.g., Rosati, 2017b; Rosati et al., 2007; Rosati & Hare, 2012; Wobber et al., 2010). Some studies have tested multiple species to understand the evolution of intelligence. For example, a study of 23 primates showed that species with greater dietary breadth, but not those living in larger groups, exhibited greater motor inhibitory control (MacLean et al., 2014), whereas another study tied inhibitory control to fission-fusion social systems (Amici et al., 2008). However, these studies focused primarily on motor inhibition, whereas research from cognitive science and neurobiology has shown that cognitive control is a multidimensional set of processes that also includes flexible updating and planning (Diamond, 2013; Friedman & Miyake, 2017; Völter et al., 2018).

Here, we aimed to bridge this gap by examining the evolution of cognitive control across primates varying in social and ecological complexity. We developed a battery of cognitive tasks measuring motor inhibition, delay of gratification, short-term memory, and shifting, which have been identified as the core, partially dissociable components of executive function in studies of adult humans and children (Diamond, 2013; Friedman & Miyake, 2017; Karr et al., 2018; Miyake et al., 2000; Wiebe et al., 2011). To implement these tasks, we drew on the methods of recent work that used a battery of tasks to assess multiple aspects of cognition in tandem on animals (e.g., Fichtel et al., 2020; Herrmann et al., 2007; Joly et al., 2017; Schmitt et al., 2012). These tasks have also been well validated in prior studies of nonhuman primates (Amici et al., 2008, 2010; Deaner et al., 2006; MacLean et al., 2014; Rosati et al., 2007). We then used this battery to examine cognitive control in primates with clear variation in both social and ecological characteristics. Importantly, both the social- and ecological-intelligence hypotheses provide plausible pathways for the emergence of cognitive control: Flexible adoption of new behavioral strategies could provide an advantage in ecological contexts, such as by allowing individuals to adjust to changing environmental circumstances (MacLean et al., 2014), but also in social contexts, by allowing individuals to deal with a shifting social landscape caused by others’ unpredictable behaviors (Amici et al., 2018).

**Statement of Relevance**

Cognitive control is a set of regulatory cognitive mechanisms that underpin flexible, intelligent behavior in humans and other animals. Capacities for cognitive control vary across species, but why these differences have evolved is unclear. Most comparative work to date has used brain size as a proxy for cognition, limiting our understanding of the evolution of specific cognitive skills. We therefore tested lemurs from four species on a novel battery of tasks tapping into multiple components of cognitive control, and we then evaluated whether these species’ social system or their ecological characteristics predicted enhanced cognitive control. We found that ecological characteristics rather than social complexity best predicted cognitive control across multiple components. These results indicate that species’ feeding ecology plays a crucial role in shaping cognitive evolution, in contrast to prevailing views that social complexity is the primary driver of intelligent behavior.

In a preregistered study, we examined cognitive control in four lemur species. Lemurs are an important taxonomic group for understanding cognitive evolution because they exhibit high levels of evolutionary diversity in both social and ecological features, even in closely related species (Fichtel & Kappeler, 2010; Richard & Dewar, 1991; Rosati, 2017b). We specifically selected lemur species that exhibit targeted, independent variation in both social and ecological characteristics to test alternative pathways for the emergence of cognitive control. Ruffed lemurs (*Varecia* species) are among the most highly frugivorous of lemurs, with diets that can exceed 90% fruit (MacLean et al., 2014; Vasey, 2005). In contrast, Coquerel’s sifakas (*Propithecus coquereli*) are obligate folivores (leaf eaters) with specialized dentition and gut structure for digestion of fibrous leaves (Campbell et al., 2000; Greene et al., 2018). Whereas fruits are a spatially and temporally variable resource, leaves are homogeneously distributed and thus foraging for them is less cognitively demanding (Milton, 1981; Rosati, 2017b). Yet both of these taxa live in medium-sized family groups (ruffed lemurs: mean group size = 6.1; sifakas: mean group size = 5.4; MacLean et al., 2009, 2014, 2013) and show similar social characteristics. Conversely, both mongoose lemurs (*Eulemur monogoz*) and ring-tailed lemurs (*Lemur catta*) exhibit intermediate diets with a mixture of fruit and leaves (MacLean et al., 2014; Ossi & Kamilar, 2006; Sauther et al., 1999), but the social structure of both types of lemur differ. Ring-tailed lemurs have some of the largest group sizes among lemurs (mean group size = 15.6; MacLean et al., 2014, 2013), with complex dominance hierarchies absent in other lemurs (Sauther et al., 1999). Mongoose lemurs, in contrast, live in small, pair-bonded groups (Ossi & Kamilar, 2006). Species who live in such larger, complex social groups experience greater social challenges related to tracking multiple individuals, assessing others’ dominance, and competing or cooperating with others (Byrne & Whiten, 1988; Dunbar, 1998).

We used this data to evaluate the two main hypotheses for the evolution of intelligence. The social-intelligence hypothesis predicted that ring-tailed lemurs, who live in larger groups with dominance hierarchies, would show enhanced cognitive control compared with other species, especially pair-bonded mongoose lemurs. The ecological-intelligence hypothesis, in contrast, predicted that frugivorous ruffed lemurs, who feed on variable fruit resources, would consistently show higher cognitive control, particularly in contrast to the highly folivorous sifakas. Because the social- and ecological-intelligence hypotheses are not mutually exclusive, an additive effect here predicted that both ruffed lemurs and ring-tailed lemurs would exhibit higher cognitive control than the other species.

## Method

### Subjects

We tested 39 lemurs living at the Duke Lemur Center (for subject information, see Table S1 in the Supplemental Material available online). We assessed four taxonomic groups: ruffed lemurs (*Varecia* species, *n* = 10), Coquerel’s sifakas (*Propithecus coquereli*, *n* = 10), ring-tailed lemurs (*Lemur catta*, *n* = 10), and mongoose lemurs (*Eulemur mongoz*, *n* = 9). Ruffed lemurs consisted of both red-ruffed and black-and-white-ruffed lemurs, but we collapsed analyses across both groups given their socioecological similarity and classification as subspecies until recently (Mittermeier et al., 2008). Our sample included all the individuals available for testing who completed the battery; two additional subjects (one sifaka and one mongoose lemur) initiated the battery but failed to reach the predetermined criterion for inclusion in several tasks or stopped participating over several days. All tests were voluntary: Lemurs were never deprived of food, had ad libitum access to water, and could stop participating at any time. The lemurs had little or no prior experience in relevant cognitive tasks such as those used here (see Table S1). All behavioral tests were approved by Duke University’s Institutional Animal Care and Use Committee (Protocol No. A268-16-12).

### General procedure

**Fig. 1.**
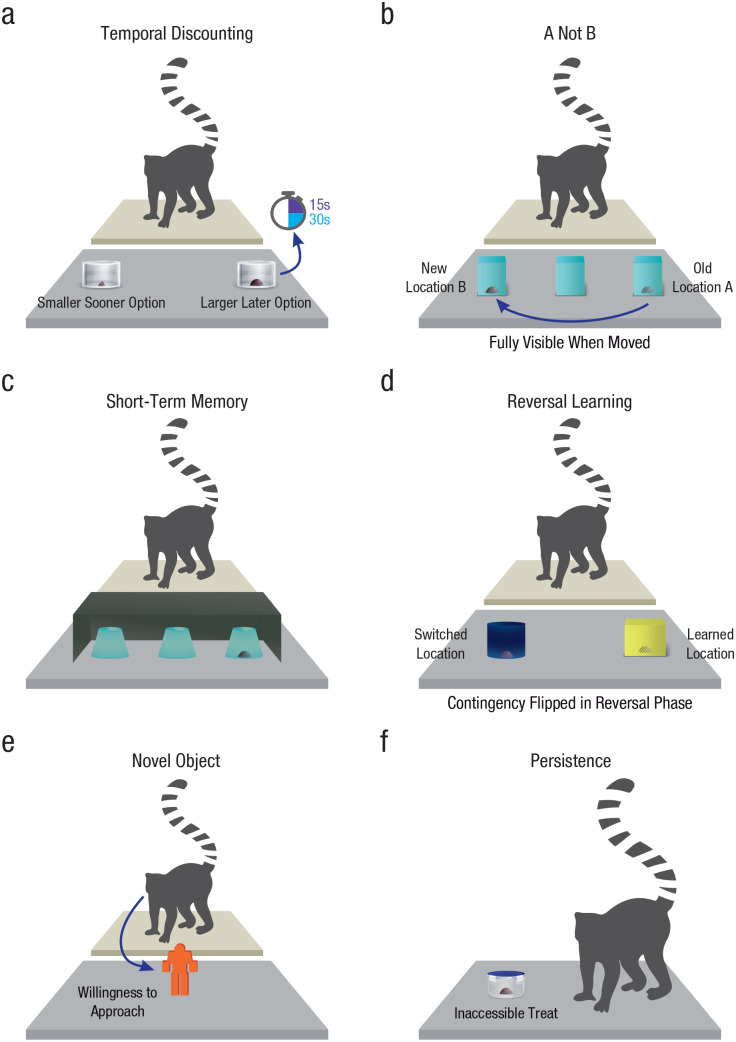
Lemur cognitive-control battery. In the temporal-discounting task (a), lemurs chose between a smaller, immediate reward and a larger, delayed reward available after either a 15-s or 30-s delay. In the A-not-B task (b), lemurs had to inhibit a prepotent motor response to access a reward. In the short-term-memory task (c), lemurs had to recall the location of hidden food after a short delay. In the reversal-learning task (d), lemurs first learned that one location contained food and then had to update contingencies. In the novel-object task (e), lemurs could approach and investigate novel stimuli. In the persistence task (f), lemurs could attempt to access inaccessible food.

Lemurs completed a battery of six well-validated tasks that assessed multiple core aspects of cognitive control (see Fig. 1): A temporal-discounting task assessed the ability to delay gratification (Rosati et al., 2007; Stevens, 2014), an A-not-B task assessed motor inhibition (Amici et al., 2008; MacLean et al., 2014), a short-term-memory task assessed the ability to hold information in mind (Amici et al., 2010; Rosati & Hare, 2012), a reversal-learning task assessed the ability to shift responses when contingencies change (Deaner et al., 2006; Wobber et al., 2010), and, finally, the novel-object task and persistence tasks both assessed individual variation in temperament (Herrmann et al., 2007; Wobber et al., 2014) that could influence cognitive performance.

In the basic procedure for the cognitive tasks, an experimenter sat across from the lemur at a sliding table and placed options containing hidden treats on the table; lemurs could indicate their choice by either touching or approaching one of the containers. All lemurs completed the tasks in the same order, typically completing one session per day and at most two tasks with a break in between. In some tasks, subjects had to first meet criteria on a pretest to demonstrate basic comprehension of the setup before proceeding to the main test, and they could repeat sessions until they passed before proceeding. If the subject did not choose one of the options within 15 s when responses involved making choices, the trial was stopped and repeated up to a maximum of four times. If the subject continued to not make a choice, the session was halted and repeated on a different day (for detailed testing procedures, see the Supplemental Material).

### Specific task procedures

#### Temporal-discounting task

The ability to delay gratification for future rewards is a key component of human executive functions (Diamond, 2013; Rosati, 2017a). To assess this ability in lemurs, we used a task in which they made decisions between a smaller option available immediately (1/16 of a grape or a peanut, depending on the species-typical diet) and a larger option available after either a 15-s or 30-s delay (1/2 of a grape or peanut; following work of Rosati et al., 2018). Subjects first completed a number-pretest session in which there was no delay attached to the larger reward to ensure that they could discriminate these quantities and preferred the larger amount when there was no delay in receiving it. Next, they completed two test sessions in which the delay associated with the larger option varied (15-s or 30-s delay). In each test session, lemurs first completed eight exposure trials (only one option available at a time) to introduce the rewards and delay contingencies. Then they completed 10 test trials in which they made choices between the smaller and larger reward (see Fig. 1a; see also Video S1 at https://doi.org/10.5061/dryad.6hdr7sr2k). The side of the delayed option on the table was counterbalanced and quasirandomized across trials (no more than three trials in a row on the same side), with a 30-s intertrial interval. We measured the lemurs’ choices for the larger reward.

#### A-not-B error task

The ability to inhibit ineffectual motor responses is another key component of executive functions (Diamond, 2013; Friedman & Miyake, 2017; Rosati, 2017a). To assess this, we used an A-not-B task, in which lemurs had to resist searching for food in a previous hiding location when the food reward was visibly moved to a new location (following methods from work of MacLean et al., 2014). Here, the experimenter put a food reward under one of three containers. Lemurs were allowed to retrieve the reward under that container three times, to develop the prepotent response. On the fourth test trial, lemurs watched as the reward was first hidden under the same container (container A) but then was moved to a different container on the other side of the table (container B; see Fig. 1b; see Video S2 at https://doi.org/10.5061/dryad.6hdr7sr2k). The same procedure was repeated three more times with visually distinct sets of containers and different hiding locations (different in color and shape; see Fig. S2 in the Supplemental Material), for a total of four test trials. The order of presentation of the container sets was fixed across subjects, and the initially baited container (left or right) was counterbalanced across trial blocks and subjects. We measured lemurs’ choices of the correct container.

#### Short-term-memory task

The ability to recall and manipulate information in the mind is another key component of executive functions (Diamond, 2013; Friedman & Miyake, 2017). To test this, we used a task in which lemurs had to recall the location of hidden food over short time intervals. Lemurs were presented with three identical containers on a sliding table. The experimenter placed a piece of food under one container in full view of the subject, and the lemur could then choose one container either immediately (no-delay trials) or after a 5-s delay in which the lemur’s view was blocked by an occluder (delay trials; see Fig. 1c; see also Video S3 at https://doi.org/10.5061/dryad.6hdr7sr2k). Although this task does not require retaining information while performing a secondary task, as is the case for many measures of working memory used with humans (Engle et al., 1999), this kind of setup is commonly used with nonhuman primates (Amici et al., 2010; Many Primates et al., 2019; Rosati & Hare, 2012) and was designed to capture species variation in lemurs’ cognition while avoiding floor effects in these species due to difficult task demands. In the task, lemurs first completed three familiarization trials in which they could immediately retrieve the food reward from containers; they then completed three blocks of trials, each consisting of three delay trials followed by one no-delay trial (for a total of 12 trials). The correct location was counterbalanced and quasirandomized (no more than two trials in a row with same location) across trials. We measured lemurs’ choices for the correct cup.

#### Reversal-learning task

The ability to flexibly update responses is another key component of executive functions (Diamond, 2013; Friedman & Miyake, 2017; Rosati, 2017a). To test this, we used a reversal-learning task in which lemurs had to switch their responses when reward contingencies changed. Lemurs were presented with two containers, differing in shape and color, on a sliding table. They initially learned that a food reward was hidden under one of the containers (unique in color and always appearing on the same side), whereas the other was always empty. Once lemurs consistently selected the baited container, the rules were switched, and the food reward was hidden under the container that was previously empty (see Fig. 1d; see also Video S4 at https://doi.org/10.5061/dryad.6hdr7sr2k). In the session, lemurs first completed two exposure trials, in which the experimenter put the food under the correct learning-phase container in full view of the subjects. Then they completed at least six (out of a maximum of 10) learning trials in which that container was baited behind an occluder. Once lemurs consistently chose the baited container (chose the correct location in six consecutive trials), they completed 10 test trials in which the reward contingencies were switched. The assignment of the baited side and of the corresponding container for the learning phase was counterbalanced across subjects. We measured lemurs’ choices for the correct container.

#### Novel-object task

We also measured two aspects of temperament in the lemurs because responses to novel or difficult situations may constrain cognitive control. For example, species might show poorer performance if they are more neophobic or less motivated to participate (Schubiger et al., 2020). To assess their responses to novelty, we showed lemurs a series of novel stimuli (following work of Herrmann et al., 2011). On each trial, one experimenter centered the lemur approximately 1.2 meters away from the table, and another experimenter placed the stimuli on the table (see Fig. 1e; see also Video S5 at https://doi.org/10.5061/dryad.6hdr7sr2k). Each lemur was presented with four stimuli in a fixed order: (a) baseline with table only, (b) baseline with a person sitting at the table, (c) novel stationary object, and (d) novel moving object (see Fig. S5 in the Supplemental Material). On each trial, we measured how long the lemur spent in close proximity to the stimuli.

#### Persistence task

In the second temperament task, we presented lemurs with an inaccessible food reward to measure their motivation to retrieve it. First, one experimenter positioned a clear box containing a piece of food on a table inside the lemur’s room. For two consecutive solvable trials, the box’s lid was left unsealed so the box could be easily opened to retrieve the food. In the third, unsolvable trial, the lid was closed so it was impossible for the lemurs to open it (see Fig. 1f; see also Video S6 at https://doi.org/10.5061/dryad.6hdr7sr2k). We measured how long subjects manipulated the box when attempting to retrieve the food; the maximum time allowed was 3 min.

### Data coding and analysis

All tasks were videotaped, and a coder blind to the study’s hypotheses coded at least 20% of tasks with high reliability (Cohen’s κ > .97 for all choice tasks, Pearson’s *r* = .99 for latency measures; for all coding details, see the Supplemental Material). The study design and statistical-analysis approach were preregistered (https://aspredicted.org/iv9tb.pdf). We used generalized linear mixed models to analyze trial-by-trial responses and compare species’ performance for most tasks (for details, see the Results section as well as the Supplemental Material). Models always included subject as a random factor to account for repeated trials when relevant, as well as age, sex, and trial number; we also accounted for pretest performance and the number of sessions each individual completed before reaching criterion (to account for each individual’s learning experiences when relevant for that task). Then we added species as a factor to examine evolutionary variation. We compared model fit using likelihood-ratio tests, and we computed post hoc pairwise comparisons with a Tukey correction. In a final analysis, we compared species’ integrative performance across tasks using principal components analysis (PCA).

## Results

**Fig. 2.**
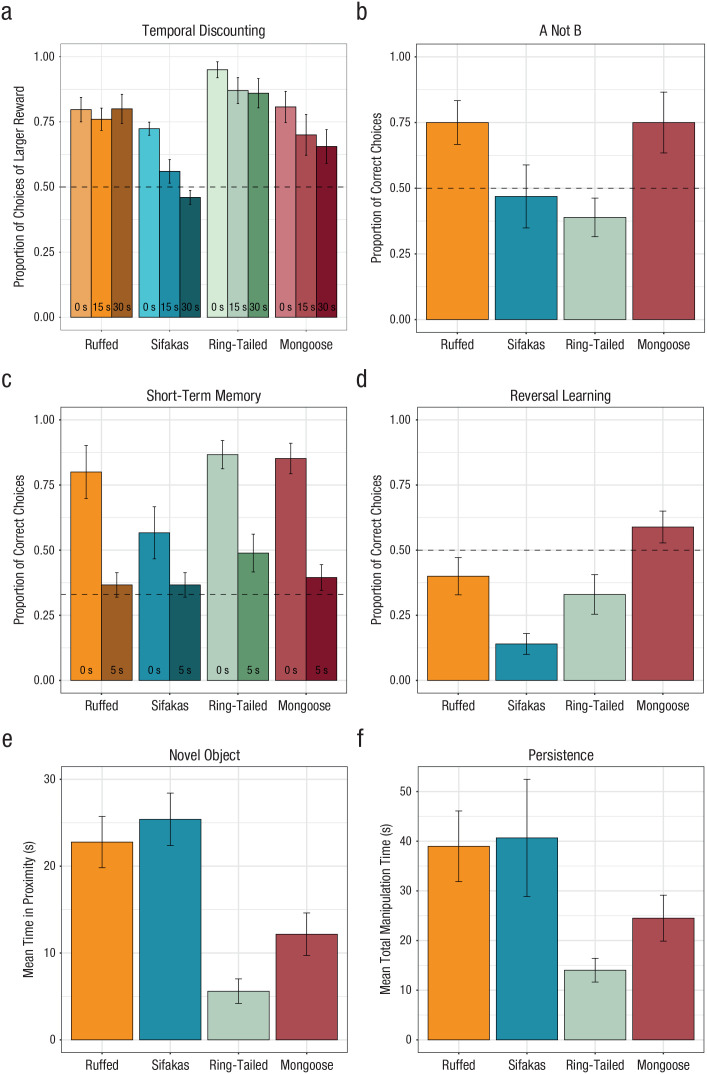
Performance of ruffed lemurs, sifakas, ring-tailed lemurs, and mongoose lemurs across tasks: (a) proportion of choices of the larger reward in each delay condition of the temporal-discounting task, (b) proportion of correct choices in the A-not-B task, (c) proportion of correct choices in each delay condition of the short-term-memory task, (d) proportion of correct choices in the reversal-learning task, (e) mean time spent in proximity to all stimuli in the novel-object task, and (f) mean time spent manipulating the box in the unsolvable trial of the persistence task. Error bars indicate standard errors, and dashed lines indicate chance performance.

**Fig. 3.**
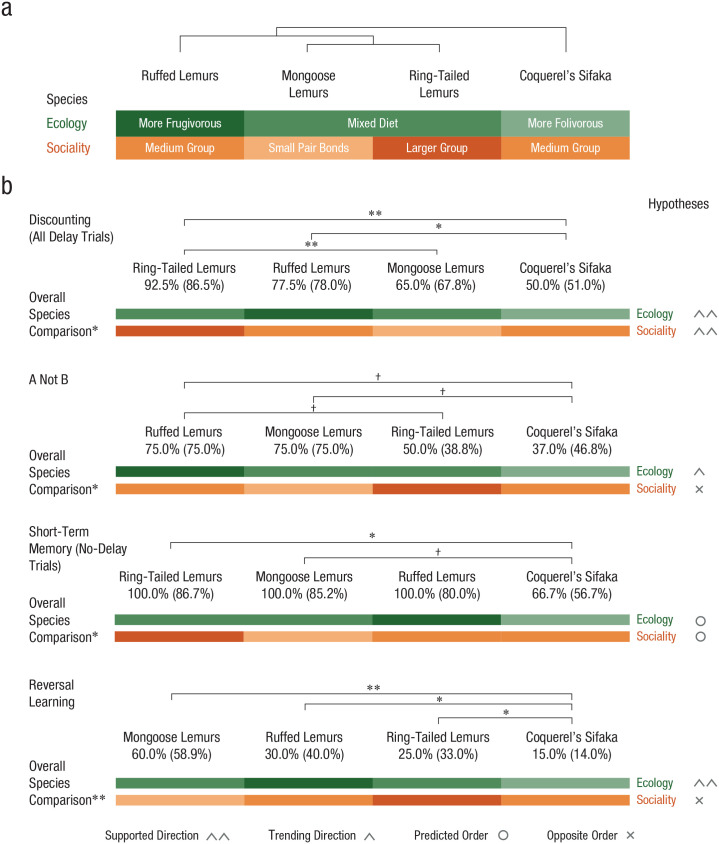
Lemur socioecology and cognitive performance. The phylogeny and socioecological characteristics of the four groups of lemur species are shown in (a). Species’ cognitive performance, ordered by their median performance, is shown in (b) for each task, along with means (in parentheses), results of overall species comparisons, results of pairwise species comparisons, and concordance of results with socioecological predictions. To the right of each graph, we indicate support for the social- and ecological-intelligence hypotheses. Support for the ecological-intelligence hypothesis is indicated when ruffed lemurs outperformed sifakas, and support for the social-intelligence hypothesis is indicated when ring-tailed lemurs outperformed mongoose lemurs. When differences across species went in the predicted direction but were not significant or showed only a trend toward significance, we use different symbols to indicate a trend or a direction in favor of or against one hypothesis. Asterisks indicate significant differences between groups (^†^*p* < .09, *p < .05, **p < .01).

An initial comparison of performance in the temporal-discounting task examining delay duration (0 s, 15 s, 30 s) indicated a linear effect of delay: As expected, lemurs chose the larger reward less often as the delay increased, χ^2^(2) = 11.97, *p* = .002 (*p* < .05 for significant comparison; for model parameters, see Table S3 in the Supplemental Material). Furthermore, all species selected the larger reward significantly more often than chance in the number pretest, in which the larger reward entailed no delay (*p* < .05 for all comparisons). This shows that lemurs accounted for the presence of delay costs in their choices. In the main analysis of the discounting task, all species except sifakas selected the longer-delayed option more often than chance in both delay conditions (*p* < .05 for significant comparisons; see the Supplemental Material for details). To compare performance across species, we included subject, age, sex, trial number, and discrimination score (the proportion of correct choices in the number pretest, to account for any variation in numerical cognition) in a base model. Adding delay (15 s and 30 s) in the second model did not improve model fit, χ^2^(1) = 0.88, *p* = .347, indicating that lemurs responded similarly to delay conditions. Adding species, however, significantly improved model fit, χ^2^(4) = 14.56, *p* = .006 (see Fig. 2a; for parameters, see Table S4 in the Supplemental Material); post hoc comparisons showed that ring-tailed lemurs were more willing to wait for a larger reward than both mongoose lemurs and sifakas, and ruffed lemurs were more willing to wait than sifakas (*p* < .05 for significant comparisons; for details, see Fig. 3 and the Supplemental Material). These results support both the social- and ecological-intelligence hypotheses, as the more socially complex ring-tailed lemurs outperformed mongoose lemurs, and the more ecologically complex ruffed lemurs further outperformed sifakas.

In the A-not-B task, only ruffed lemurs preferentially selected the correct container at an above-chance level on test trials (*p* < .05), and performance of mongoose lemurs showed a trend toward doing so (*p* = .07); performance of ring-tailed lemurs and sifakas did not differ from chance (for details, see Fig. 2b and the Supplemental Material). To compare performance, we included subject, age, sex, trial number, and number of sessions each individual needed to complete before reaching criterion (to account for any individual variation in performance on familiarization trials) in a base model. Inclusion of species improved model fit, χ^2^(3) = 11.52, *p* = .009 (see Fig. 2b; for parameters, see Table S5 in the Supplemental Material). Post hoc comparisons revealed a trend for mongoose lemurs and ruffed lemurs to outperform sifakas (*p* = .09) and for ruffed lemurs to outperform ring-tailed lemurs (*p* = .06; see Fig. 3). This pattern is most in line with predictions of the ecological-intelligence hypothesis, as the ruffed lemurs tended to outperform the sifakas as well as the ring-tailed lemurs, but does not support the predictions of the social-intelligence hypothesis, as the ring-tailed lemurs did not outperform the mongoose lemurs.

In the short-term-memory task, all species selected the correct option at an above-chance level on no-delay trials (*p* < .05 for all comparisons), whereas in the delay trials, only ring-tailed lemurs’ performance was marginally above chance (*p* = .056: see the Supplemental Material for details). In the comparison of species, a base model accounting for subject, age, sex, trial number, number of sessions each individual needed to complete before reaching criterion (to account for experience in familiarization trials) and condition (immediate or delayed choice) confirmed that lemurs performed better when they could retrieve the food immediately. Adding species as a predictor in a second model showed a trend toward significantly improved model fit, χ^2^(3) = 6.35, *p* = .096, as did adding the interaction between species and condition, χ^2^(6) = 11.44, *p* = .076 (see Fig. 2c; for parameters, see Table S6 in the Supplemental Material). We further explored this result by analyzing lemurs’ performance only in the no-delay trials, given the lemurs’ poor performance in delay trials. We found that adding species as a predictor in a second model improved model fit, χ^2^(3) = 10.67, *p* = .014. Post hoc comparisons in this analysis revealed that ring-tailed lemurs outperformed sifakas (*p* < .05; see Fig. 3) and that mongoose lemurs showed a trend toward outperforming sifakas (*p* = .072). No other differences among species were found. Overall, this suggests that lemurs have fairly poor short-term memory overall, but there was no evidence in support of the social-intelligence hypothesis. Rather, the folivorous sifakas’ relatively low performance was most in line with the ecological-intelligence hypothesis.

In the reversal-learning task, we focused on lemurs’ ability to update their responses in the reversal phase. Overall, sifakas and ring-tailed lemurs performance remained at below-chance levels across reversal trials, whereas mongoose and ruffed lemurs performed at chance (*p* ≤ .05 for significant comparisons); note that individuals were expected to start below chance and improve over reversal trials in this task as they learned the correct response after the contingency switches. Here, the base model accounted for subject, age, sex, trial number, and learning trials needed to reach criterion (to account for individual variation in initial learning experience) and showed that performance overall improved over reversal trials, as expected. Inclusion of species improved model fit, χ^2^(3) = 19.08, *p* < .001 (see Fig. 2d; for parameters, see Table S7 in the Supplemental Material). Post hoc comparisons indicated that sifakas showed worse performance than all other species (*p* < .05 for all comparisons; see Fig. 3). No other differences among species were found. Overall, this supports the ecological-intelligence hypothesis—given that the frugivorous ruffed lemurs as well as the intermediate mongoose and ring-tailed lemurs all outperformed the folivorous sifakas. However, there was again no support for the predictions of the social-intelligence hypothesis, as performance of the mongoose lemurs and the ring-tailed lemurs did not differ.

Some proposals suggest that temperament may constrain performance in cognitive tasks (Herrmann et al., 2007; Schubiger et al., 2020), so we next assessed whether species-level variation in temperament could explain the pattern of results in the cognitive-control measures. In the novel-object task, lemurs spent the longest overall amount of time in proximity to a novel stationary object (compared with a moving object or baseline trials; see the Supplemental Material). Adding species to a base model including subject, age, sex, and trial type improved model fit, χ^2^(3) = 26.75, *p* < .001 (see Fig. 2e; for parameters, see Table S8 in the Supplemental Material); sifakas spent more time near the novel items than both ring-tailed and mongoose lemurs (*p* < .05 for both comparisons), and ruffed lemurs spent more time than ring-tailed lemurs (*p* < .05). No other differences among species were found. Additional comparisons showed that these results were driven primarily by differences in responses to the stationary and moving objects (see the Supplemental Material). In the persistence task, the inclusion of species in a base model accounting for sex and age also improved model fit, χ^2^(3) = 14.65, *p* = .002 (see Fig. 2f; for parameters, see Table S9 in the Supplemental Material); sifakas and ruffed lemurs spent more time manipulating the box compared with ring-tailed lemurs (*p* < .05 for significant comparisons). No other differences among species were found. Overall, these results indicate that sifakas, the species with the worst cognitive performance in general, were quite bold and willing to engage in tasks, and they generally had similar temperament outcomes to the more cognitively successful ruffed lemurs. Thus, neophobic responses or low levels of motivation did not seem to constrain performance across the cognitive-control tasks.

**Fig. 4.**
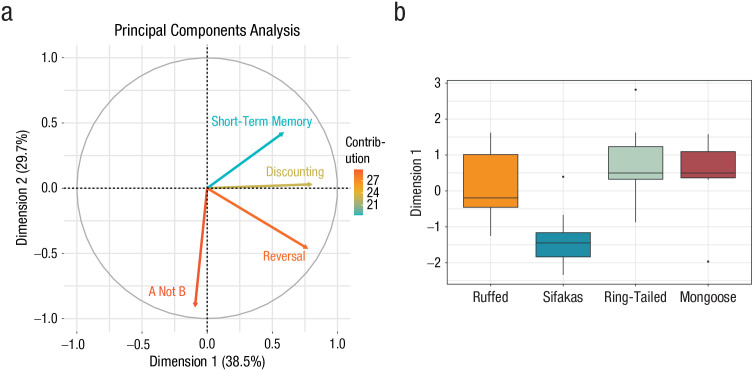
Results of the principal components analysis of cognitive-control tasks. The contribution of each of the tasks to the two unrotated dimensions extracted from the analysis is shown in (a). Dimension (Component) 1 explains 38.5% of the variance in task performance. Dimension (Component) 2 explains 29.7% of the variance. Arrows indicate the contributions of each task to these dimensions. The box plots (b) show the average summary scores for Dimension 1, separately for ruffed lemurs, sifakas, ring-tailed lemurs, and mongoose lemurs. In each box plot, the hinges indicate the lower and upper quartile, the horizontal line represents the median, and the whiskers indicate the minimum and maximum range of the analyzed data. Outliers are plotted as individual points.

As a final test of the social- and ecological-intelligence hypotheses, we implemented a PCA to extract summary scores of each species’ responses across the four cognitive-control tasks. We then compared variation in these extracted scores across the species to test for differences (for details, see the Supplemental Material). The PCA yielded two principal unrotated components that best explained variation in lemurs’ performance, on the basis of an analysis that compared eigenvalues from actual data with randomly resampled and simulated correlation matrices (Budaev, 2010). The temporal-discounting, short-term-memory, and reversal-learning tasks positively loaded on the first component (explaining 38.5% of the variance), whereas the A-not-B task loaded on the second component (accounting for 29.7%; see Fig. 4a; for loadings of each task in the component, see Table S11 in the Supplemental Material). We then compared species’ scores on these two dimensions and found that the sifakas were significantly different from the other species in Dimension 1, reflecting an overall lower performance in the cognitive-control tasks (*p* < .05 for all comparisons; see Fig. 4b; see also Table S12 in the Supplemental Material). The ring-tailed lemurs also showed a pattern more like sifakas on Dimension 2, which primarily tracks A-not-B performance (see Table S13 in the Supplemental Material). Thus, results from the PCA complement the results seen in each individual task: We found that the least ecologically complex species (sifakas) showed the worst performance across tasks on both dimensions. In contrast, there was again limited or even negative support for the social-intelligence hypothesis, given that the most socially complex species (ring-tailed lemurs) actually showed a decrement in performance on Dimension 2.

## Discussion

We examined the evolution of cognitive control in lemurs using a novel battery of cognitive tasks, and we found that ecological complexity more consistently predicted cognition across species than did social complexity across contexts. In particular, the sifakas—the only obligate folivore in our study—showed the worst performance across multiple measures and specifically were often outperformed by the highly frugivorous ruffed lemurs in the temporal-discounting, A-not-B, and reversal-learning tasks. Crucially, these species exhibit similar social systems but differ in the complexity of their diets. In contrast, there was no consistent pattern of variation related to sociality. Ring-tailed lemurs, who live in large social groups with dominance hierarchies, were quite successful at delaying gratification and successful to a lesser extent in the short-term-memory task. However, they did not otherwise consistently outperform the pair-bonded mongoose lemurs or the other species in general; indeed, the mongoose lemurs showed stronger performance in both the A-not-B task and the reversal-learning task. The PCA further indicated that the sifakas showed worse performance overall across both extracted dimensions and that the ring-tailed lemurs also had worse performance in Dimension 2. Crucially, the novel-object and persistence measures indicated that these patterns of cognitive performance were not due to temperamental constraints. Thus, our study indicates that ecology rather than sociality plays a more fundamental role in shaping cognitive control in lemurs.

These results align with prior work showing that spatial memory and decision-making vary according to ecology in primates (e.g., De Petrillo & Rosati, 2020; MacLean et al., 2014; Rosati, 2017b; Rosati et al., 2014; Stevens, 2014). Yet these findings also contrast with those of prior work showing that ring-tailed lemurs outperform mongoose lemurs in social cognition (MacLean et al., 2008, 2013). One possibility is that these different cognitive domains evolve independently: Social capacities such as perspective taking may depend on social complexity, whereas abilities such as spatial memory that are needed to locate food depend on ecological complexity (Rosati, 2017b; Sandel et al., 2011). Importantly, cognitive control is often considered a domain-general process (Diamond, 2013; Friedman & Miyake, 2017), so our results indicate that some domain-general abilities may also be shaped primarily by ecological pressures. Yet an important consideration is that our battery, in adapting core measures of executive function from studies of humans (Diamond, 2013; Friedman & Miyake, 2017; Karr et al., 2018; Miyake et al., 2000), implemented tasks that in some ways resemble foraging tasks, as animals made decisions in nonsocial contexts to obtain a reward. Thus, an important question for future work concerns understanding the evolution of executive functions across different contexts. There has been some work implementing social versions of cognitive-control problems (e.g., social-reversal learning: Wobber et al., 2010; social-inhibitory control: Reddy et al., 2015), but there has been little attempt to assess whether animals actually show different skill levels in social than in foraging contexts. In fact, some evidence suggests that several primate species show largely similar inhibitory-control capacities across these contexts (Amici et al., 2008, 2018). Future comparisons of other components of executive function across both foraging and social contexts is therefore important to understand the evolution of cognitive control.

Another important question concerns the mechanistic basis of cognitive control in primates. In adult humans, different components of inhibition, working memory, and shifting represent distinct, dissociable components of executive control (Friedman & Miyake, 2017). Our PCA suggests that some aspects of cognitive control (e.g., delay of gratification, short-term memory, and reversal learning) exhibit shared variation in lemurs. However, future work could build on this by implementing a battery with multiple measures capturing each component and using factor analysis to infer the latent structure of cognition (Herrmann, Hérnández-Lloreda, et al., 2010; MacLean et al., 2017). Given that interrelationships between cognitive skills can differ across species, this approach is also important to understand how cognition evolves (Herrmann, Hérnández-Lloreda, et al., 2010; MacLean et al., 2017; Völter et al., 2018). A related question concerns the links between these skills and the brain regions that support them. In humans, core components of executive function are dissociable not just in behavioral measures but also in their neural basis. Prior work has linked inhibitory control in primates to absolute brain size (MacLean et al., 2014), and a crucial next step is examining whether distinct components of cognitive control are linked to distinct brain regions, as in humans.

A final point concerns the fundamental problem of how to best conceptualize ecological and social complexity across species. Here, we took the approach of comparing species that differed across broad, widely accepted metrics of ecological and social complexity—degree of frugivorous versus folivorous diet and the size and complexity of social groups. Yet there are alternative approaches to this problem. Although frugivory diets are generally considered more ecologically complex than folivory diets in primates (DeCasien et al., 2017; Milton, 1981; Rosati, 2017b), other metrics such as caching, niche specialization, or dietary breadth are sometimes used with other species (MacLean et al., 2014; Henke-von der Malsburg et al., 2020). Yet it is worth noticing that obligate folivores such as the sifakas are underrepresented in comparative work, despite being critical for testing socioecological hypotheses (Tan et al., 2014). Similarly, although primate species with larger group sizes (Dunbar, 1998) or many differentiated social relationships (Bergman & Beehner, 2015) are generally considered to be more complex, other features such as social tolerance, fission–fusion social groups, or pair bondedness may also be important (Amici et al., 2008; Dunbar & Shultz, 2007; Joly et al., 2017). However, we note that the mongoose lemurs did not consistently outperform the other species, so we also did not find support for the pair-bond hypothesis here. Overall, this highlights the importance, but also the difficulty, of indexing social and ecological complexity in a manner that is generally useful but also appropriate for the species under consideration (Henke-von der Malsburg et al., 2020).

In conclusion, we examined cognitive control across lemurs using measures of temporal discounting, motor inhibition, short-term memory, and reversal learning. Our results show that cognitive-control capacities consistently varied according to these species’ feeding ecology but not according to their social complexity. Thus, our findings align with accumulating evidence that ecological complexity can be an important driver of both cognitive and brain evolution in lemurs (MacLean et al., 2009; Rosati et al., 2014) and across primates in general (DeCasien et al., 2017; MacLean et al., 2014; Rosati, 2017b). Further, this provides converging support for the proposal that ecological processes were important in human cognitive evolution as well (e.g., González-Forero & Gardner, 2018). Making robust inferences about the evolution of cognition requires integrating both approaches from cognitive science concerning how to best capture the underlying cognitive mechanisms that influence behavior, as well as assessing cognitive abilities across diverse species.

## Supplemental Material

sj-docx-1-pss-10.1177_09567976221082938 – Supplemental material for The Evolution of Cognitive Control in Lemurs Click here for additional data file. Supplemental material, sj-docx-1-pss-10.1177_09567976221082938 for The Evolution of Cognitive Control in Lemurs by Francesca De Petrillo, Parvathy Nair, Averill Cantwell and Alexandra G. Rosati in Psychological Science

## Transparency

*Action Editor:* Steven Gangestad

*Editor:* Patricia J. Bauer

*Author Contributions*

F. De Petrillo and A. G. Rosati designed the study. F. De Petrillo and P. Nair collected the data at the Duke Lemur Center. F. De Petrillo, P. Nair, and A. Cantwell coded the data, and F. De Petrillo and A. G. Rosati analyzed the data. F. De Petrillo and A. G. Rosati wrote the manuscript with input from all the authors. All the authors approved the final manuscript for submission.

**Open Practices:** Videos of all the tasks and the data for this study have been made publicly available via Dryad and can be accessed at https://doi.org/10.5061/dryad.6hdr7sr2k. The design and analysis plans for the study were preregistered on AsPredicted at https://aspredicted.org/iv9tb.pdf. This article has received the badges for Open Data, Open Materials, and Preregistration. More information about the Open Practices badges can be found at http://www.psychologicalscience.org/publications/badges.
