# Supplementary material for: The Evolution of Cognitive Control in Lemurs
Source: Psychol Sci. 2022 Jul 25;33(9):1408–22. doi: 10.1177/09567976221082938 (PMC10068506; doi:10.1177/09567976221082938)
Supplement: sj-docx-1-pss-10.1177_09567976221082938 – Supplemental material for The Evolution of Cognitive Control in Lemurs [file sj-docx-1-pss-10.1177_09567976221082938.docx]

***Supplementary Online Materials***

*The evolution of cognitive control in lemurs*

**Subjects**

We tested 39 lemurs at the Duke Lemur Center (see Table S1 for detailed subject information). This included ten Coquerel's sifakas (*Propithecus coquereli*; 6 females and 4 males; ranging from 2 to 21 years old; mean age = 7.4 years); ten ring-tailed lemurs (*Lemur catta*; 6 females and 4 males; ranging from 4 to 24 years old; mean age = 12.1 years); nine mongoose lemurs (*Eulemur mongoz*; 3 females and 6 males; ranging from 2 to 29 years old; mean age = 9.2 years); and ten ruffed lemurs (6 red ruffed lemurs *Varecia rubra* and 4 black-and-white ruffed lemurs *Varecia variegata*; 2 females and 8 males; ranging from 2 to 34 years old; mean age = 14.3 years; analyses collapse *Varecia* spp. given their overall similarity and classification as subspecies until recently [1]).

Lemurs were housed in species-appropriate social groups and had access to indoor and outdoor rooms; many could also semi-free-range in forested enclosures for more than half the year, temperatures permitting. Lemurs had *ad libitum* access to water during the sessions, were not food-restricted for testing, and were fed a species-appropriate daily diet of fruit, vegetables, leaves, and chow. All tests were voluntary: if the lemur stopped participating, the session was stopped. These lemurs had little or no prior experience in relevant cognitive tasks that involved making choices between containers to obtain food or measuring temperament as implemented in this study (see Table S1 for relevant experience by subject). An additional 2 subjects (1 sifaka and 1 mongoose lemur) were initially started in the battery but were not included in the final data set because they failed to reach criterion in several tasks or stopped participating over several days.

**General procedure**

Lemurs completed a cognitive control battery administered across 6 days of testing, with all individuals completing the tasks in the same order across days. On Day 1 they completed the novel object task, number pretest, and persistence task; on Days 2 and 3 they completed two sessions of the temporal discounting task (with different delays); on Day 4 they completed the A-not-B task; on Day 5 they completed the short term memory task; and on Day 6 they completed the reversal learning task. Each subject generally received one test session per day, with the possibility to complete a second session if they did not pass task-specific criterion or did not participate initially in the first session. In this case, there was a break of at least 15 minutes between the sessions. Lemurs could attempt the same task up to four times; lemurs completed all the tests within this time frame, with the exception of four individuals who did not meet criterion in the A-not-B task and then proceeded to the subsequent tasks.

During testing, the primary experimenter (E1) typically sat outside the room, separated from the lemur by wire mesh, and presented the task on a plastic table (length 76.2 cm, width 45.72 cm, height 49.53 cm); the lemur sat on an identical table inside their room to view stimuli and make choices. In most tasks, the experimenter placed relevant options on a sliding platform within view of the lemur, and then pushed the table forward so that lemurs could indicate their choice by touching one option, or approaching one option within 3 cm from their initial centered starting position. The experimenter looked down or at the center of the table (e.g., not at the options) to avoid any potential social cueing. A second experimenter (E2) filmed lemurs’ choices and recorded choices live on paper. All tasks were videotaped.

| Lemur | Species | | Sex | Age | Studies | Lemur | Species | Sex | Age | Studies |
| --- | --- | --- | --- | --- | --- | --- | --- | --- | --- | --- |
| Beatrice | Pc | F | | 6.6 | - | Amor Jr. | Vv | M | 6.2 | 2 |
| Elliot | Pc | M | | 6.7 | 2 | Borealis | Vr | M | 31.2 | 2,3 |
| Hostilian | Pc | M | | 3.5 | - | Buzz | Vr | M | 2.1 | 2 |
| Isabella* | Pc | F | | 4.4 | - | Comet | Vr | M | 34.7 | 3 |
| Justa | Pc | F | | 7.5 | - | Cosmo | Vv | M | 3.0 | - |
| Magdalena | Pc | F | | 2.1 | - | Kalani | Vr | M | 3.0 | 2 |
| Rupilia* | Pc | F | | 20.4 | 2 | Kizzy | Vv | F | 14.2 | 2,3 |
| Sigmund | Pc | M | | 2.7 | - | Minias | Vr | M | 25.3 | - |
| Valeria | Pc | F | | 4.5 | - | Pandora | Vr | F | 8.1 | 2 |
| Wenceslaus | Pc | M | | 4.4 | - | Rees | Vv | M | 6.2 | 2 |
| Alena* | Lc | F | | 14.3 | - | Bonita | Em | F | 3.2 | - |
| Berisades | Lc | M | | 15.3 | 3 | Carolina | Em | F | 9.3 | - |
| Fritz | Lc | M | | 17.2 | - | Duggan | Em | M | 11.3 | - |
| Hibernia | Lc | F | | 10.3 | - | Ignacio | Em | M | 4.2 | - |
| Onyx | Lc | M | | 6.2 | - | Maddie | Em | F | 13.1 | 3 |
| Persephone | Lc | F | | 14.2 | - | Mico | Em | M | 5.2 | - |
| Princess Julien | Lc | F | | 4.2 | - | Nacho | Em | M | 2.3 | - |
| Sierra Mist | Lc | F | | 11.2 | - | Oscar | Em | M | 5.2 | - |
| Teres | Lc | M | | 24.3 | 3 | Pedro* | Em | M | 29.1 | 3 |
| Thea | Lc | F | | 4.2 | - |  |  |  |  |  |

**Table S1. Subjects characteristics**. Identity, species, sex, age, and prior cognitive testing experience by individual. Sex: F = female, M = male. Species, Pc = *Propithecus coquereli*; Lc = *Lemur catta*; Vv = *Varecia variegata*; Vr = *Varecia rubra*; Em = *Eulemur mongoz*. Studies refers to subjects with experience in prior cognitive tasks involving similar testing setups, specifically De Petrillo & Rosati 2020 [2] and MacLean et al 2012 [3]. *Individuals that did not complete the full A-not-B task and were not included in the main analysis for that task; partial data was used in the PCA analysis as described below.

**Statistical analysis approach**

We conducted three main kinds of analyses, both implemented in R version 4.0.2 [4]. First, we examined whether differences in species’ socioecology predict variation in subjects’ performance in each task of the battery (see Table S2 for species performance across tasks). To analyze tasks with multiple trials, we implemented generalized linear mixed models (GLMMs) using the *glmer* function or linear mixed models using the *lmer* function, both from the *lme4* package [5]. Models included *subject* as a random intercept to account for repeated measurements within-subjects, as well as subject’s *age*, *sex,* and *trial number*. Additional predictors such as *number of sessions* needed to reach criterion were included in models as relevant for that task. For analyses of the persistence task (with only one test trial per subject), we used generalized linear models implemented with the *glm* function. Across these models, the dependent variable was whether choices were correct (cognitive tasks; binary measure) or behavioral responses (durations of response in temperament tasks). We then added *species* as a predictor to see if this improved model fit, as the main test of our hypotheses. Across analyses, we compared the fit of different models using likelihood-ratio tests [6]. Post-hoc pair-wise comparisons of model predictors were computed using the *emmeans* package [7] with a Tukey correction.

To examine relationships between tasks, we then examined whether performance on a given cognitive task predicts performance in other tasks. We first used pairwise bivariate Pearson correlations between tasks by using the *corr* package [8]. To provide positive support for null findings, we used Bayes factor (BF), by implementing the *correlationBF* and the bayesfactor function in the *BayesFactor* package [9]. Finally, we implemented a principal component analysis to detect whether performance in different tasks co-varies across individuals using the *stats* package [4].

Data files are available here:

(<https://drive.google.com/drive/folders/17x3cq2YqQ8cLa0jT5rXiX3mWrPfpM5tf?usp=sharing>).

| Species | Cognitive control tasks | | | | Temperament | |
| --- | --- | --- | --- | --- | --- | --- |
|  | Discounting  *Mean choices LL* | A-not-B  *Mean correct choice* | Memory  *Mean correct choice* | Reversal  *Mean correct choice in the reversal phase* | Novelty  *Mean time in proximity* | Persistence  *Total manipulation* |
| Ruffed | 0.78 ± 0.03 | 0.75 ± 0.08 | 0.58 ± 0.07 | 0.40 ± 0.07 | 22.77 ± 2.95 | 38.98 ± 7.11 |
| Sifakas | 0.51 ± 0.03 | 0.47 ± 0.12 | 0.47 ± 0.06 | 0.14 ± 0.04 | 25.39 ± 3.02 | 40.67 ± 11.8 |
| Ring-tailed | 0.86 ± 0.04 | 0.39 ± 0.07 | 0.68 ± 0.06 | 0.33 ± 0.08 | 5.60 ± 1.42 | 14.03 ± 2.39 |
| Mongoose | 0.68 ± 0.05 | 0.75 ± 0.12 | 0.62 ± 0.07 | 0.59 ± 0.06 | 12.16 ± 2.45 | 24.51 ± 4.62 |

**Table S2.** Species performance across tasks. Mean correct choices (SE) are indicated for each task and species.

**Temporal discounting task**

In the temporal discounting task, lemurs made a series of choices between a small piece of preferred food available immediately and a bigger piece of the same food available after a delay of either 15 or 30 seconds according to the condition. We adapted methods for this task from prior work with primates [10,11].

*Number pretest*

Before the main test sessions, subjects completed at least 10 (up to 20 maximum) quantity discrimination trials, where they could choose between the smaller and larger foods used in the discounting task (1/16 of the reward vs. ½ of the reward; grapes were used for ring-tailed, mongoose, and ruffed lemurs, whereas peanuts were used for the sifakas as this species has different dietary needs due to being folivorous). This pretest was designed to initially confirm that lemurs could discriminate between these food items, and preferred the larger amount when there was no delay to wait. The food was placed on two clear disks on opposite sides of a sliding platform (see figure S1 and Video S1). The position of the larger reward (left or right) was counterbalanced and quasi-randomized with the restriction that it could not be on the same side more than twice in a row. To proceed to the next task of the battery, lemurs had to choose the larger piece of food in 7 out of the last 10 trials.

*Test sessions*

On two subsequent days, lemurs completed the main discounting task sessions. Here, the larger food was available only after a 15s (first test session) or 30s (second test session) delay (see Video S1). Each test session comprised 18 trials each with the same structure. Lemurs first completed 8 initial *forced-choice trials* (four per option, in which only one option was available to ensure familiarity with the delays associated with each reward), followed by 10 *choice trials* where they made choices between the two options. The order of presentation of the initial forced-choice trials was counterbalanced and quasi-randomized across trials (no more than 2 trials in a row with the same assignment), so that the subject experienced each option on both the left and the right side twice. In the test trials, the side assignment of the small and large reward was also counterbalanced and quasi-randomized (no more than 3 trials in a row with the same assignment) across trials. There was a 30s inter-trial interval (ITI) between trials measured by a stopwatch, starting when the lemur placed the food in their mouth. Between trials, lemurs’ view of the table was blocked by an occluder.

*Coding and reliability*

Choices were coded live by experimenter 2. A second coder, who was blind to the hypotheses, coded choices on 100% of test trials from videotape with high agreement (Kappa = 0.97).


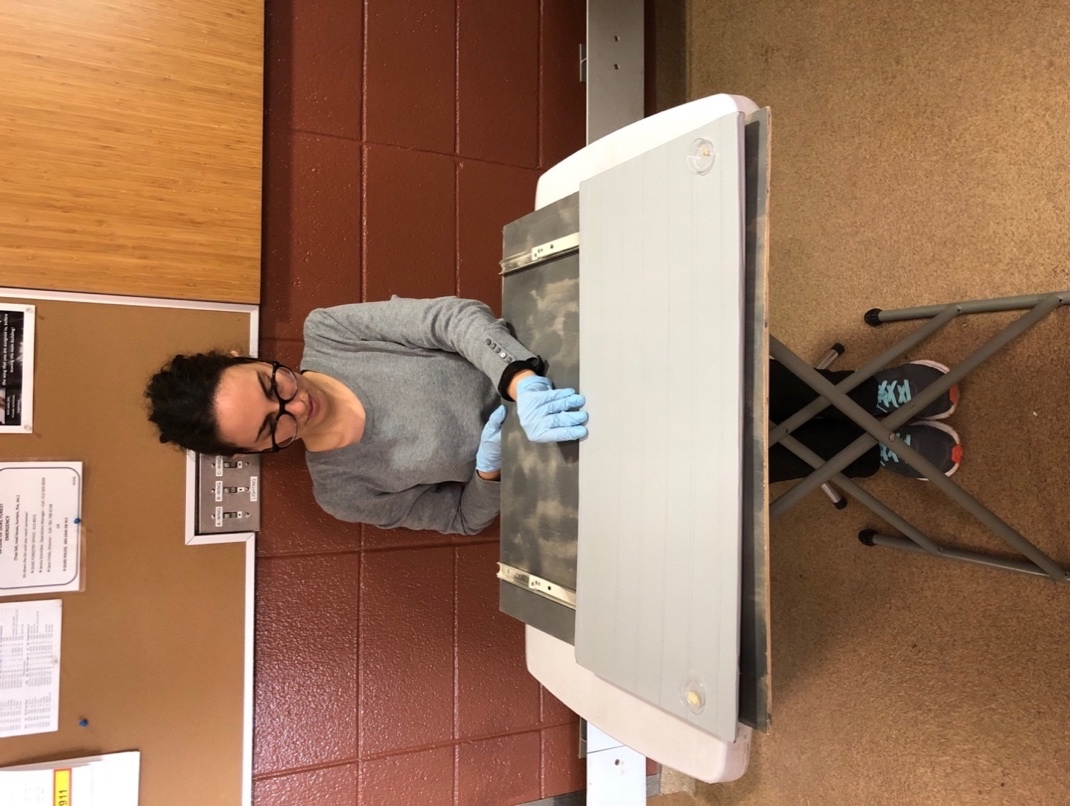


**Figure S1. Setup for the temporal discounting task.** A smaller piece of food (on the left from the experimenter’s view; 1/16 of a peanut) and a larger piece of the same food (on the right; 1/2 of a peanut) were placed on opposite sides of a sliding table inside two clear plastic disks. If lemurs selected the larger piece of food, they had to wait either 15 or 30 second delays according to the condition before having access to it (no delay was imposed in the number pretest).

*Choice analyses*

In the number pretest all species selected the larger option above chance [one-sample t-tests; *ruffed*: 0.80 ± 0.05, t_9_ = 6.33, p < 0.001; *sifakas*: 0.72 ± 0.02, t_9_ = 8.89, p < 0.001; *ring-tailed*: 0.95 ± 0.03, t_9_ =14.64, p < 0.001; *mongoose*: 0.81 ± 0.06, t_8_ = 5.14, p < 0.001]. To confirm that lemurs were sensitive to the presence of delays in the task, we examined binary trial-by-trial responses across the number pretest and discounting test sessions. To do so, we fit binomial GLMM models with the logit link function. A base model included subject’s *age*, *sex*, *trial number*, and random *subject* intercepts to account for repeated measurements within subjects. To examine whether lemurs chose the larger reward more often in the number pretest where there was no delay imposed, versus the test trials where they had to wait some delay for the larger reward, we then added *delay duration* (0s, 15s, or 30s) as an ordered factor in a second model. This improved model fit [LRT: χ^2^ =11.97, df = 2 p = 0.002; see Table S3 for full model parameters], with a significant linear effect of delay. Pairwise comparisons showed that lemurs choose the larger reward significantly more often in the 0s number pretest compared to the 15s and 30s delay [p < 0.05], indicating that lemurs were sensitive to the costs associated with waiting.

| Predictor | Estimate | S.E. | Z | *p* value |
| --- | --- | --- | --- | --- |
| Age (in years) | 0.006 | 0.019 | 0.341 | 0.733 |
| Sex (reference: females) | 0.017 | 0.324 | 0.054 | 0.957 |
| Trial Number | -0.003 | 0.022 | -0.158 | 0.874 |
| Delay Linear | -0.455 | 0.186 | -2.446 | **0.015** |
| Delay Quadratic | 0.165 | 0.154 | 1.071 | 0.284 |

**Table S3. Predictors of performance in the number pretest and temporal discounting task.** Parameters from the best-fitting model comparing lemurs’ choice when they faced different delays.

To compare delay of gratification across species, we then examined the main discounting sessions where there was a delay associated with the larger option (15s or 30s). Overall, all species except sifakas selected the larger delayed option above chance in both delay conditions [one-sample t-tests*; ruffed 15s delay*: 0.76 ± 0.04, t_9_ = 6.09, p < 0.001; *ruffed 30s delay:* 0.80 ± 0.05: t_9_ = 5.38, p < 0.001; *sifakas 15s delay*: 0.56 ± 0.04, t_9_ = 1.33, p = 0.217, n.s.; *sifakas 30s delay*: 0.46 ± 0.03, t_9_ = -1.5, p = 0.168, n.s.; *ring-tailed 15s delay*: 0.87 ± 0.05, t_9_ = 7.47, p < 0.001; *ring-tailed 30s delay*: 0.86 ± 0.06, t_9_ = 6.41, p < 0.001; *mongoose 15s delay*: 0.70 ± 0.08, t_8_ = 2.56, p = 0.034; *mongoose 30s delay*: 0.66 ± 0.06, t_8_ = 2.40, p = 0.043].

To compare performance across species, we implemented a base model including *age*, *sex*, *trial number* (1-10)*, subject* as a random factor*,* and finally that subject’s *number discrimination* *score* (the proportion of correct choices in the number pretest, to account for any individual variation in number performance). This showed that lemurs with higher discrimination scores chose the larger delayed option more often in the test sessions. In a second model we then added *delay condition* (15s and 30s) as a predictor, which did not improve model fit [LRT: χ^2^ = 0.88, df = 1; p = 0.347], indicating that lemurs responded similarly for both delays. In a third model we then added *species* as predictor, which significantly improved model fit [χ^2^ = 14.56, df = 4, p = 0.006; see Table S4 for parameters of the best fitting model]. Post-hoc comparisons showed that ring-tailed lemurs outperformed mongoose lemurs and sifakas [*ring-tailed/mongoose:* p = 0.03; *ring-tailed/sifakas:* p = 0.001], and ruffed lemurs outperformed sifakas [p = 0.04]. No other differences among species were found [*ring-tailed/ruffed*: p = 0.38; *ruffed/mongoose*: p = 0.58].

| **Predictor** | **Estimate** | **S.E.** | **Z** | ***p* value** |
| --- | --- | --- | --- | --- |
| Age (in years) | 0.007 | 0.018 | 0.396 | 0.692 |
| Sex (reference: females) | 0.260 | 0.293 | 0.888 | 0.374 |
| Trial Number | 0.006 | 0.030 | 0.194 | 0.846 |
| Discrimination score | 0.893 | 1.154 | 0.774 | 0.439 |
| Delay (reference: 15s) | -0.161 | 0.170 | -0.943 | 0.345 |
| Species Mongoose (reference: Sifakas) | 0.613 | 0.397 | 1.543 | 0.123 |
| Species Ring-tailed (reference: Sifakas) | 1.820 | 0.489 | 3.720 | **< 0.001** |
| Species Ruffed (reference: Sifakas) | 1.107 | 0.423 | 2.618 | **0.008** |

**Table S4. Predictors of performance across species in the temporal discounting task.** Parameters from the best-fitting model predicting lemurs’ choice in the temporal discounting test sessions.

**A-not-B task**

In the A-not-B task, lemurs experienced reaching for food in one location several times, and then were tested on their ability to inhibit this prepotent reach when the reward was visibly moved to a new location. We adapted methods in this task from prior work with primates [12].

*Methods*

Lemurs completed one session comprising 4 blocks of trials. In each block, lemurs were presented with three identical containers in a row on a sliding platform, of which only one was baited with food (different, visually-distinct containers were used on each block; see Figure S2). In each block, they first completed at least 3 (maximum up to 6) *familiarization trials* in which a piece of food was consistently placed under one of the containers (Container A; either the left or right container) in view of the lemur, and the lemur could then choose this location. The experimenter always closed the container under which the food was positioned first, and then closed the other containers from left to right. After having successfully retrieved the food three consecutive times, lemurs completed one *test trial* for that block in which they initially saw the food being hidden under the same container (Container A), but then watched as the food was moved to another container at the other end of the table (Container B; see Video S2). Each block of trials consisted of a new set of visually-distinct containers with different colors and shapes; the containers were always presented in the same order across blocks for all individuals. Lemurs experienced a 3-minute break between blocks to differentiate these periods. The side assignment for container A was counterbalanced across trial blocks and subjects (half of subjects experienced that container A was on the right for the first two sets, and on the left on the second two sets; this was reversed for the other subjects).

If lemurs did not choose correctly within a total of 6 familiarization trials for a given block, the session was ended and repeated the next day. The session could be repeated up to a maximum of four sessions before proceeding with the other tasks in the battery. Lemurs needed between 1 and 4 sessions to complete the task (mongoose lemurs needed on average 2 sessions to reach criterion; ring-tailed lemurs needed on average 1.7 sessions; ruffed lemurs needed on average 1.8 sessions; and sifakas needed on average of 1.7 sessions). Four lemurs (2 sifakas, 1 ring-tailed lemur and 1 mongoose lemur) failed to reach these criteria, so they were not included in the main analyses of this task. Their partial data was included in the PCA analysis, so we ran the analyses of the main task including their partial data to check that we found the same pattern of results as in the main analyses (see below).


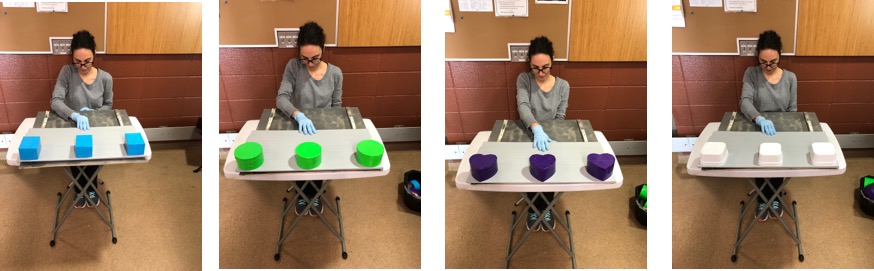


**Figure S2. Setup for the A-not-B error task.** In each block of trials, lemurs first experienced that one of three identical containers (either the left or right container) consistently contained food. On the test trial for that block, the food was first placed in that container and then visibly moved to a new container on the other side of the table. Across different blocks, lemurs experienced visually-distinct sets of containers.

*Coding and reliability*

Choices were coded live by experimenter 2. A second coder, who was blind to the hypotheses, coded choices on 100% of trials of lemurs’ final session from videotape (with high agreement, Kappa = 0.99).

*Main choice analyses*

We analyzed performance in the test trials. While ruffed lemurs selected the correct container above chance on test trials [one-sample t-tests; 0.75 ± 0.08, t_9_ = 3, p = 0.015], and mongoose lemurs trended to do so [0.75 ± 0.12, t_7_ =2.16, p = 0.07], ring-tailed lemurs [0.39 ± 0.07, t_8_ = -1.51, p = 0.17, n.s.] and sifakas [0.47 ± 0.12, t_7_ = -0.26, p = 0.80, n.s.] did not differ from chance.

To compare performance across species, we implemented a base model including *age*, *sex*, *trial block* (1-4), *subject* as a random factor, and *number of* *sessions to criterion* (to account for any individual variation in performance on familiarization trials). Adding *species* significantly improved model fit [χ^2^ = 11.52, df = 3, p = 0.009; see Table S5 for parameters]. Post-hoc comparisons showed a trend for both ruffed lemurs and mongoose lemurs to outperform sifakas [*ruffed/sifakas*: p = 0.089; *mongoose/sifakas*: p = 0.086], and for ruffed lemurs to also outperform ring-tailed lemurs [p = 0.062]. No other differences among species were found [*ring-tailed/sifakas*: p = 0.10; *ring-tailed/mongoose*: p = 0.12; *ruffed/mongoose*: p = 0.10].

| **Predictor** | **Estimate** | **S.E.** | **Z** | ***p* value** |
| --- | --- | --- | --- | --- |
| Age (in years) | -0.050 | 0.031 | -1.595 | 0.111 |
| Sex (reference: females) | 0.816 | 0.479 | 1.706 | 0.088 |
| Number Sessions Criterion | -0.406 | 0.249 | -1.626 | 0.104 |
| Trial block | 0.106 | 0.174 | 0.608 | 0.543 |
| Species Mongoose (reference: Sifakas) | 1.596 | 0.678 | 2.354 | **0.019** |
| Species Ring-tailed (reference: Sifakas) | 0.114 | 0.632 | 0.180 | 0.857 |
| Species Ruffed (reference: Sifakas) | 1.719 | 0.736 | 2.336 | **0.019** |

**Table S5. Predictors of performance across species in the A-not-B task.** Parameters from the best-fitting model predicting lemurs’ choices on test trials.

*Choice analyses with partial data included*

To confirm that our results were not affected by the four subjects that did not reach the criterion, as well to validate the use of partial data in the principal component analysis, we repeated this same analysis procedure also including the partial data available for these subjects. We used the session in which each individual completed the majority of the test trials, which corresponded to the third session for three individuals and the fourth session for the final individual. Overall, we found the same general results as in the main analyses: adding *species* as a factor increased model fit [χ^2^ = 11.52, p = 0.009]. Post-hoc comparisons confirmed our previous results: ruffed lemurs and mongoose lemurs both trended to outperform sifakas [p = 0.09 for all comparisons], and ruffed lemurs trended to also outperform ring-tailed lemurs [p = 0.06].

**Short term memory task**

In the short term memory task, lemurs had to recall the location of hidden food following a short delay. We adapted methods in this task from prior work with primates [13-15].

*Methods*

Lemurs completed one session where they were presented with 3 identical cups in a row on the sliding table, and had to recall which container held hidden food (Figure S3). Lemurs first completed at least 3 (maximum 6) initial *familiarization trials* to demonstrate they understood the task setup. Here E1 placed a piece of food under one of the cups in full view of the subject, and immediately after the lemur was allowed to choose; subjects could proceed to the test trials only if they chose the correct cup on three consecutive familiarization trials. As in the A-not-B task, E1 always closed the cup under which the food was positioned first, and then closed the others from left to right. If lemurs did not choose correctly within a total of 6 familiarization trials the session was repeated, up to a maximum of four sessions. Lemurs needed 1-3 sessions to reach the criterion (mongoose lemurs needed on average 1 session to reach criterion, ring-tailed lemurs needed on average 1.2 sessions, ruffed lemurs needed on average 1.3 sessions, and sifakas needed on average 1.3 sessions).

In the main test, lemurs then completed three blocks of trials, each comprised of three *delay trials* (food occluded for 5s before choice) followed by 1 *no-delay trial* (immediate choice, like the initial familiarization trials). In the delay trials, their view of the table was occluded by a black carboard screen (see Video S3). The position of the food was counterbalanced across the three locations and quasi-randomized over trials (no more than 2 trials in a row with the same location).


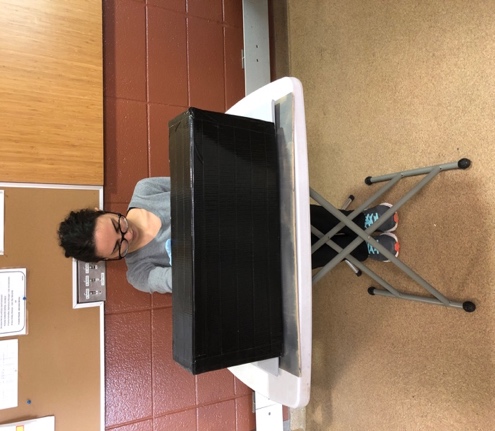

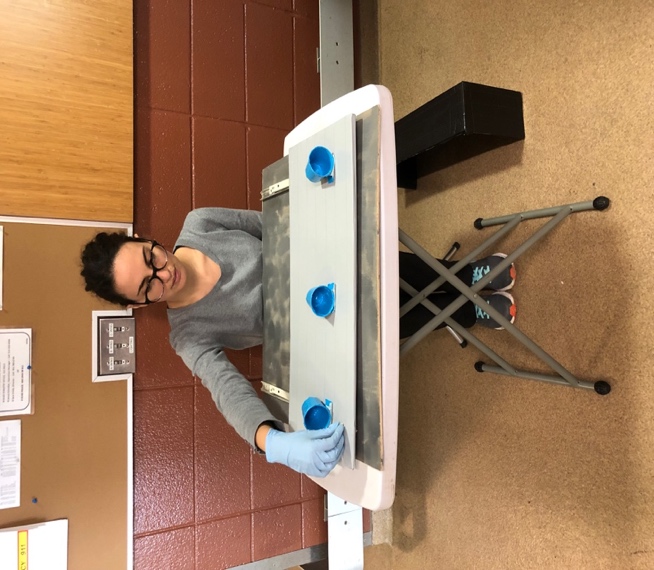

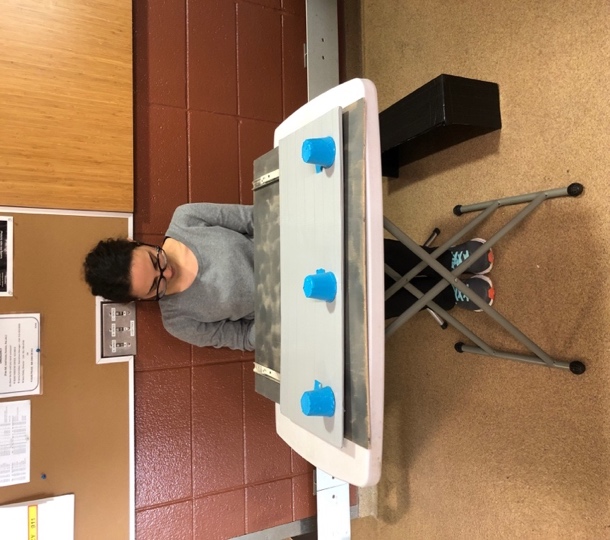


a)

b)

c)

**Figure S3. Setup for the working memory task.** In delay trials, **(**a) a food reward was placed under one of the cups in full view of the subject; (b) their view of the table was blocked for 5s by an occluder; and (c) the subject could choose. In no-delay trials, subjects could choose immediately after baiting.

*Coding and reliability*

Choices were coded live by experimenter 2. A second coder, who was blind to the hypotheses, coded choices on 100% of trials of lemurs’ test session from videotape with high agreement (Kappa = 0.99).

*Choice analyses*

We examined lemurs’ performance in the main test trials. In the no-delay trials, all species selected the corrected option above chance [one-sample t-tests; *ruffed*: 0.80 ± 0.10, t_9_ = 4.61, p = 0.001; sifakas: 0.57 ± 0.10, t_9_ = 2.37, p = 0.042; *ring-tailed*: 0.87 ± 0.05, t_9_ = 9.86, p < 0.001; *mongoose*: 0.85 ± 0.06, t_8_ = 8.91, p < 0.001; note that chance here is 33%], whereas in the delay trials only ring-tailed lemurs trended to choose it above chance [*ring-tailed*: 0.49± 0.07, t_9_ = 2.19, p = 0.056], whereas the performance of the other species was at chance [*ruffed*: 0.37 ± 0.05, t_9_ = 0.78, p = 0.45, n.s.; *sifakas*: 0.37 ± 0.05, t_9_ = 0.78, p = 0.45, n.s.; *mongoose*: 0.39 ± 0.05, t_8_ = 1.32, p = 0.22, n.s.].

To compare performance across species, we implemented a base model including *age*, *sex*, number *sessions needed to reach criterion*, *trial number* (for that trial type), *subject* as a random factor*,* and *trial type* (delay or no-delay trial). This confirmed that overall lemurs performed better in the no-delay trials. Inclusion of *species* in a second model only trended to improve model fit [χ^2^ = 6.35, df = 3, p = 0.096], and adding the interaction between species and trial type in a third model also trended to improve fit [χ^2^ = 11.44, df= 6, p = 0.076; see Table S6 for parameters of the best fitting model]. As reported in the main text, post-hoc comparisons showed that, in the no-delay trials specifically, mongoose and ring-tailed lemurs trended to outperform sifakas [*mongoose/sifakas*: p = 0.094; and ring-tailed/sifakas: p = 0.068]. No other differences among species were found [*ring-tailed/mongoose*: p = 0.10; *ring-tailed/ruffed:* p = 0.94; *ruffed/mongoose*: p = 0.98; ruffed/sifakas: p = 0. 17].

| **Predictor** | **Estimate** | **S.E.** | **Z** | ***p* value** |
| --- | --- | --- | --- | --- |
| Age (in years) | -0.002 | 0.015 | -0.147 | 0.883 |
| Sex (reference: females) | -0.238 | 0.219 | -1.087 | 0.277 |
| Number Sessions Criterion | -0.037 | 0.231 | -0.158 | 0.874 |
| Trial Number | -0.028 | 0.042 | -0.672 | 0.502 |
| Trial Type (reference: no delay) | -0.735 | 0.447 | -1.643 | 0.100 |
| Species Mongoose (reference: Sifakas) | 1.549 | 0.669 | 2.314 | **0.021** |
| Species Ring-tailed (reference: Sifakas) | 1.622 | 0.663 | 2.447 | **0.014** |
| Species Ruffed (reference: Sifakas) | 1.235 | 0.606 | 2.038 | **0.042** |
| Trial Type * Species Mongoose (reference: Sifakas) | -1.369 | 0.730 | -1.876 | **0.060** |
| Trial Type * Species Ring-tailed (reference: Sifakas) | -1.108 | 0.721 | -1.537 | 0.124 |
| Trial Type * Species Ruffed (reference: Sifakas) | -1.124 | 0.666 | -1.690 | 0.091 |

**Table S6. Predictors of performance across species in the short term memory task.** Parameters from the best-fitting model predicting lemurs’ choice in the main test trials.

Finally, as a follow-up we further compared performance across species only in the no-delay trials, given that there was greater variation in performance in this subset of trials as most of the individuals exhibited fairly poor performance in the delay trials. Here, the inclusion of *species* in a second model did improve model fit [χ^2^ = 10.67, df = 3, p = 0.014]. Post-hoc comparisons showed that ring-tailed lemurs outperformed sifakas [p < 0.05], and that mongoose lemurs trended to outperform sifakas [p = 0.07].

**Reversal learning task**

In the reversal learning task, lemurs first learned that one container had a food reward, and then had to update their responses when the correct response flipped. We adapted methods in this task from prior work with primates [16-17].

*Methods*

Two containers of different colors and shapes were positioned on opposite sides of the sliding table (see Figure S4). Lemurs first learned that one container always provided a food reward, whereas the other was always empty. Then, the contingencies were switched (reversal learning) and the food was always located under the opposite container (see Video S4). In one session lemurs completed up to 4 *exposure trials*, up to 10 possible *learning trials*, and 10 *test trials*. In the 4 *exposure trials,* food was positioned under one of the containers in full view of the subject to introduce lemurs to the basic procedure. If they chose the correct container on two consecutive exposure trials, they then proceeded to the *learning trials* (if not, they repeated the session the next day). In *learning trials*, the same container was baited as in exposure trials, but here the baiting was completed behind an occluder. Lemurs had to select the correct container 5 out of the 6 previous learning trials (up to a maximum of 10 in one day); if lemurs failed to reach this criterion, they repeated the session. Finally, they completed 10 *test trials* where the contingencies of the rewarded option were switched, such that the food was placed under the opposite container. The assignment of the baited side and of the corresponding container (blue vs. yellow) for the learning phase was counterbalanced across subjects. One ring-tailed lemur was tested with a white box (rather than yellow) because he consistently refused to touch the yellow container.


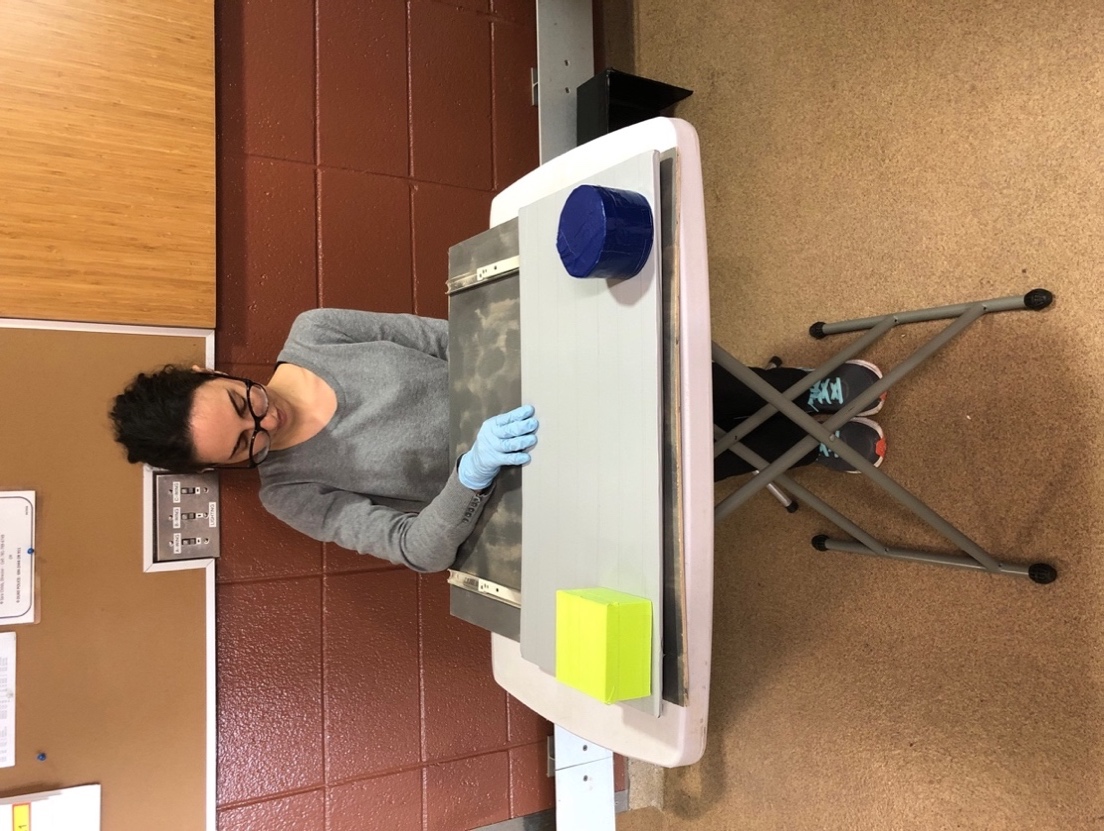


**Figure S4. Setup used in the reversal learning task.** Lemurs first learned that the food was under the blue container, then in the test trial the contingencies switched, and the reward was positioned under the yellow container.

*Coding and reliability*

Choices were coded live by the experimenter 2. A second coder, who was blind to the hypotheses, coded choices on 100% of trials of lemurs’ final session from videotape with high agreement (Kappa = 0.99).

*Choice analyses*

We examined lemurs’ performance in the reversal trials. Across all trials, sifakas and ring-tailed lemurs remained below chance [*sifakas*: 0.14 ± 0.04, t_9_ = -9, p < 0.001; *ring-tailed*: 0.33 ± 0.08, t_9_ = -2.23, p = 0.05]. Note that lemurs should start out choosing the incorrect option following learning trials, so this means these species did not adjust their responses over trials. Mongoose lemurs’ and ruffed lemurs’ performance, in contrast, was at chance level over the entire set of reversal trials [one-sample t-tests; *mongoose*: 0.59 ± 0.06, t_8_ = 1.45, p = 0.18, n.s.; *ruffed*: 0.40 ± 0.07, t_9_ = -1.40, p = 0.19, n.s.].

To compare performance across species, a base model including *age*, *sex*, number of *learning trials needed to reach criterion* (to account for individual variation in initial learning), *subject* as a random factor, and *trial number* (1-10) showed that lemurs’ performance increased across the trials, as expected. Inclusion of *species* in a second model improved model fit [χ^2^ = 19.08, df = 3, p < 0.001; see Table S7 for parameters of the best fitting model]. As reported in the main text, post-hoc comparison indicated that sifakas showed the worst performance of all species [*ruffed/sifakas*: p = 0.02; *mongoose/sifakas*: p < 0.01; *ring-tailed/sifakas*: p = 0.045]. No other differences among species were found [*ring-tailed/mongoose*: p = 0.12; *ring-tailed/ruffed:* p = 0.98; *ruffed/mongoose*: p = 0.21].

| **Predictor** | **Estimate** | **S.E.** | **Z** | ***p* value** |
| --- | --- | --- | --- | --- |
| Age (in years) | -0.010 | 0.021 | -0.511 | 0.609 |
| Sex (reference: females) | 0.355 | 0.364 | 0.974 | 0.330 |
| Number Learning Trials | 0.055 | 0.127 | 0.434 | 0.664 |
| Trial Number | 0.232 | 0.045 | 5.138 | **<0.001** |
| Species Mongoose (reference: Sifakas) | 2.456 | 0.535 | 4.594 | **<0.001** |
| Species Ring-tailed (reference: Sifakas) | 1.359 | 0.521 | 2.608 | **0.009** |
| Species Ruffed (reference: Sifakas) | 1.548 | 0.542 | 2.854 | **0.004** |

**Table S7. Predictors of performance across species in the reversal learning task.** Parameters from the best-fitting model predicting lemurs’ choice in the reversal learning task. Post-hoc comparisons between species are reported in the main text.

**Novel object task**

In this temperament task, lemurs could approach a variety of stimuli to assess their reactions to novelty. We adapted methods in this task from prior work with primates [10,18].

*Methods*

As in the cognitive tasks, two adjacent plastic tables were placed on either side of the testing room’s wire mesh. The table inside the lemur’s room was positioned inside the enclosure and connected to the cage using zip-ties and the experimental table was located in the hallway. On the lemur’s table, a blue line indicated a zone of proximity to the objects that was later used for video coding (40.6 cm distance from the object). The test was filmed with two cameras, one filming the whole cage, and the other angled from above to film the 106.5 x 111.7 cm area in front of the table.

On each trial lemurs were attracted to a starting position (using a small piece of food) that was 61 cm away from the table along the wire-mesh of the room. To allow subjects to clearly see the objects, the lemurs were centered on the wire mesh at approximately the same height of the table. Once the lemur was centered, Experimenter 1 said start, and placed the relevant object on the exterior table on relevant trials (see Video S5). Each trial lasted 60s, and we measured how much time lemurs spent near the stimuli.

Lemurs completed 4 trials in the following order: (1) *Baseline*: No experimenters or objects present; (2) *Person-only*: E1 sat at the experimenter’s table outside the lemur’s enclosure with their head facing downwards; (3) *Stationary novel object*: E1 sat at the table and placed a plastic orange toy on the table, centered against the mesh (4) *Moving novel object*: E1 sat at the table and moved a remote-controlled toy car back and forth on the external table using a hidden controller.

*
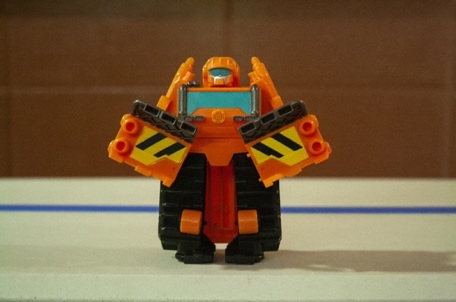

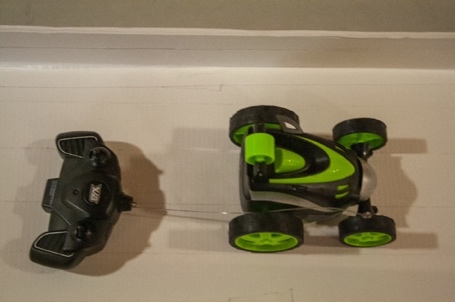
*

a)

b)

**Figure S5. Stimuli for the novel object task.** (a) The stationary novel object. (b) The moving object. In trials 3 and 4 lemurs could approach these items.

*Coding and reliability*

To evaluate lemurs’ response to novelty, we measure the amount of time the lemurs spent in the area on the table close to the object (e.g., demarcated by blue tape). One coder scored all trials from video and another independent coder scored 20% for reliability. Inter-rater reliability was high (Pearson’s R = 0.99).

*Proximity analyses*

We analyzed total duration of time spent near the stimuli using linear mixed models. As reported in the main text, a base model accounted for *sex*, *age*, *subject* as a random factor, and *trial type* (baseline, person, stationary object and moving object). This indicated that lemurs spent more time in proximity to the stationary object than in the other trials [*stationary/baseline*: p < 0.001; *stationary/person*: p = 0.057; *stationary/moving*: p = 0.027]. Other comparisons were not significant [*baseline/person*: p = 0.47; *baseline/moving*: p = 0.65; person/moving: p = 0.10]. In a second model we added *species* as a predictor, which improved model fit [χ^2^ = 26.75, df = 3, p < 0.001; see Table S8 for parameter of this model]. Sifakas spent more time near the novel items than did ring-tailed and mongoose lemurs [*sifakas/ring-tailed*: p < 0.001; *sifakas/mongoose*: p = 0.007], and ruffed lemurs spent more time near the novel object than ring-tailed lemurs [p < 0.003]. No other differences among species were found [*ring-tailed/mongoose*: p = 0.43; *ruffed/mongoose*: p = 0.12; ruffed/sifakas: p = 0.64]. In a third model, we then added an interaction between *trial type X species*, which further improved model fit [ χ^2^ =26.71, p = 0.002]. Post-hoc comparisons showed that sifakas spent more time in proximity than ruffed and ring-tailed lemurs during the baseline trial [*sifakas/ruffed*: p = 0.048; *sifakas/ring-tailed*: p = 0.051]. No other differences among species were found in this trial [*sifakas/mongoose*: p = 0.90; *ruffed/mongoose*: p =0.22; *ruffed/ring-tailed*: p = 0.10; *mongoose/ring-tailed*: p = 0.25]. In the *person-only trial*, sifakas also spent more time in proximity than ring-tailed and mongoose lemurs [*sifakas/ring-tailed*: p = 0.01; *sifakas/mongoose*: p = 0.03]. No other differences were found in this trial [*sifakas/ruffed*: p = 0.76; *ruffed/mongoose*: p =0.27; *ruffed/ring-tailed*: p = 0.15; *mongoose/ring-tailed*: p = 0.10]. In the *stationary novel object trial*, sifakas spent more time in proximity than mongoose and ring-tailed lemurs [*sifakas/mongoose*: p = 0.32; *sifakas/ring-tailed*: p = 0.002], and ruffed lemurs spent more time than ring-tailed lemurs [p = 0.05]. No other differences were found in this trial [*sifakas/ruffed*: p = 0.73; *ruffed/mongoose*: p =0.30; *mongoose/ring-tailed*: p = 0.85]. Finally, in the *moving object* trial sifakas spent more time in proximity than ring-tailed lemurs, and ruffed lemurs spent more time than both mongoose and ring-tailed lemurs [*sifakas/ring-tailed*: p = 0.01; *ruffed/mongoose*: p < 0.001; *ruffed/ring-tailed*: p < 0.001]. No other differences were found in this trial [*sifakas/ruffed*: p = 0.37; *sifakas/mongoose*: p =0.10; *mongoose/ring-tailed*: p = 0.85].

| **Predictor** | **Estimate** | **S.E.** | **df** | **t** | ***p* value** |
| --- | --- | --- | --- | --- | --- |
| Age (in years) | 0.190 | 0.176 | 33.00 | 1.079 | 0.288 |
| Sex (reference: females) | 2.748 | 3.063 | 33.00 | 0.897 | 0.376 |
| Trial Type Person (reference: baseline) | 4.736 | 3.282 | 114.00 | 1.443 | 0.152 |
| Trial Type Stationary (reference: baseline) | 13.112 | 3.282 | 114.00 | 3.995 | **< 0.001** |
| Trial Type Moving (reference: baseline) | 3.815 | 3.282 | 114.00 | 1.163 | 0.247 |
| Species Mongoose (reference: Sifakas) | -14.529 | 4.168 | 33.00 | -3.486 | **0.001** |
| Species Ring-tailed (reference: Sifakas) | -20.912 | 4.088 | 33.00 | -5.115 | **< 0.001** |
| Species Ruffed (reference: Sifakas) | -5.081 | 4.295 | 33.00 | -1.183 | 0.245 |

**Table S8. Predictors of responses across species in the novel object task.** Parameters from the second model predicting lemurs’ response to novelty across trials.

Finally, we further checked that our results were not driven by subjects that never approached the area of the table in proximity of the stimuli. To do so, we conducted the same set of analyses, but excluded those trials where the duration code was 0s. This supplementary analysis confirmed the same general pattern of results: inclusion of species as a predictor in the model improved model fit [χ^2^ = 14.82, p = 0.002]: sifakas spent more time near the novel items than did ring-tailed [p = 0.005] and mongoose lemurs [p = 0.054]. No other differences were found [*sifakas/ruffed*: p = 0.35; *ruffed/mongoose*: p =0.70; *ruffed/ring-tailed*: p = 0.22; *mongoose/ring-tailed*: p = 0.77]. Here, it was not possible to examine responses to the moving object trials specifically because that was where the most individuals did not approach.

**Persistence task**

In this temperament task, lemurs were presented with an ‘unsolvable’ task in order to assess their persistence to obtain a reward. We adapted methods in this task from prior work with primates/dogs [19-21].

*Methods*

In this task, lemurs were presented with a clear box with a lid that contained a piece of food. After initial experiences where the box could be easily opened, in the final test trial the lid was sealed so it could not be opened (see Figure S6). The box was placed on a plastic table inside the lemur’s room. In the initial *solvable trials*, the lid was placed on top of the box without sealing it in order for the lemur to easily retrieve the food inside. Subjects had a maximum of 60 seconds to open the box (see Video S6). If subjects failed to open the box in the first trial, E1 removed the lid in full view of the subject and let the lemur retrieve the food. Then, the trial was repeated. If the subject failed again, this procedure could be repeated a maximum of two more times. After successfully completing two *solvable* trials, the box was sealed in the single *unsolvable trial*. This trial lasted for 3 minutes, and we measured how long the lemurs persisted in manipulating the box.

*Coding and reliability*

We coded the total amount of time subjects spent touching the box or with their face 2.5 cm from the box. One coder scored all trials from video and another independent coder scored 20% for reliability. Inter-rater reliability was high (Pearson’s R = 0.99).


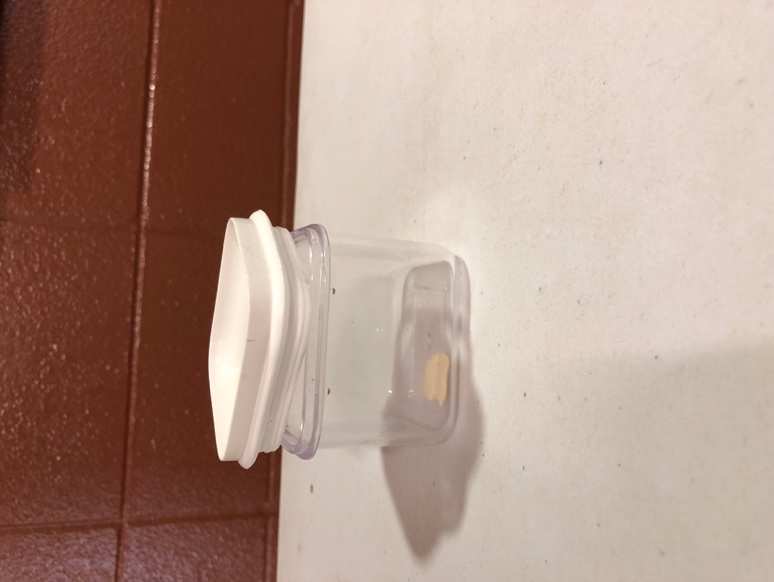

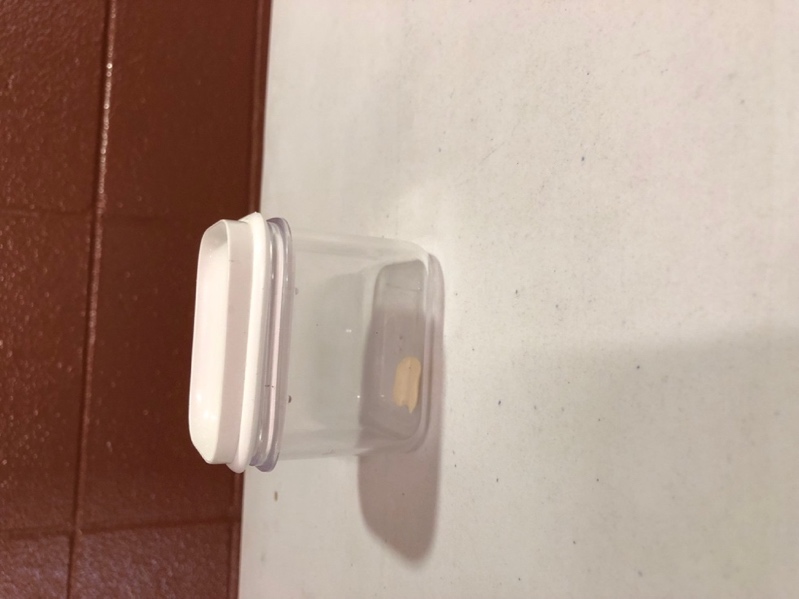


**Figure S6. Setup used in the persistence task.** In the initial solvable trials (on the left) lemurs could easily open the box by pushing off the lid. In the unsolvable trial (on the right) the lid was sealed so the lemurs could not open it to retrieve the treat inside.

*Total manipulation time analyses*

Since the persistence data were right skewed, we analyzed time spent manipulating the box using generalized linear models implemented with gamma family (identity link) to account for this data distribution [22]. As reported in the main text, inclusion of *species* as a predictor to a base model accounting for *sex* and *age* improved model fit [χ^2^ =14.65, df = 3, p = 0.002; see Table S9 for parameters of the model]. Sifakas and ruffed lemurs spent more time manipulating the box compared to ring-tailed lemurs [*sifakas/ring-tailed*: p = 0.02; *ruffed/ring-tailed*: p = 0.02]. No other differences among species were found [*ruffed/sifakas*: p = 0.10; *sifakas/mongoose:* p = 0.43; *ruffed/mongoose*: p = 0.49; *ring-tailed/mongoose*: 0.31].

| **Predictor** | **Estimate** | **S.E.** | **t** | ***p* value** |
| --- | --- | --- | --- | --- |
| Age (in years) | 0.119 | 0.355 | 0.335 | 0.740 |
| Sex (reference: females) | -4.168 | 5.230 | -0.797 | 0.432 |
| Species Mongoose (reference: Sifakas) | -15.705 | 10.383 | -1.513 | 0.140 |
| Species Ring-tailed (reference: Sifakas) | -26.916 | 9.485 | -2.838 | **0.008** |
| Species Ruffed (reference: Sifakas) | -1.601 | 12.258 | -0.131 | 0.897 |

**Table S9. Factors influencing lemurs’ performance in the persistence task.** Parameters from the second model predicting lemurs’ persistence. Post-hoc comparisons between species are reported in the main text.

**Comparisons of performance across tasks**

To investigate lemur species’ performance holistically across tasks as well the relationships between performance across tasks, we conducted a series of bivariate correlation followed by a principal component analysis. We did not account for sex or age in these analyses as we did not find major effects in the individual tasks. For these analyses, we included the following measures for each individual:

- Discounting: proportion of choices of the larger delayed reward averaged across the two delay conditions (15s and 30s)
- A-not-B: proportion of choices of the correct location in the four test trials
- Short term memory: proportion of correct choices averaged across all the experimental trials (0s and 5s)
- Reversal: proportion of choices of the correct location in the test trials, i.e. when the reward contingencies are switched
- Novel object: mean amount of time spent in proximity to the items collapsed across the four trials (baseline, person, static and moving objects)
- Persistence task: total amount of time spent manipulating the box during the 3 minutes of the unsolvable trial

*Bivariate correlations*

We first used pairwise bivariate Pearson correlations between tasks, and then examined Bayes Factors (BFs) to provide positive support for null findings. The Bayesian hypothesis test for correlations allows a quantification of the null hypothesis that the correlation between pairs of variables is zero [23]. When we find support for correlation between two tasks, we report BF10, which quantifies evidence for the alternative hypothesis. Whereas when we did not find a correlation, we reported the BF01 which quantifies evidence for the null hypothesis relative to the alternative hypothesis (where BF01 = 1/BF10).

We found three main results. First, we found a negative correlation between individuals’ willingness to wait to obtain a larger reward in the discounting task, and their willingness to approach in the novelty task (n = 39, r_p_ = - 0.37, p = 0.02, BF_10_ = 3.66) and willingness to search in the persistence task (n = 39, r_p_ = -0.45, p = 0.004, BF_10_ = 13.84). That is, individuals who were more interested in novelty and more persistent were less likely to delay gratification. Second, we also found a negative relationship between reversal learning and response to novelty (n = 39, r_p_ = -0.37, p = 0.02, BF_10_ = 3.74): individuals who were bolder in the novel object task were less successful at updating responses in the reversal learning task. Finally, we found a positive correlation between temporal discounting performance and cognitive flexibility in the reversal learning task (n = 39, r_p_ = 0.40, p = 0.01, BF_10_ = 6.18): individuals who were better able to delay gratification were also more successful at updating responses. BF_10_ values for these correlations indicated moderate or strong support for the hypothesis that there is a relationship between tasks. We found no correlations between the other tasks, and Bayes Factors values for these correlations indicated some support for the null hypothesis of no relationship between tasks (see Table S10).

|  | Discounting | A-not-B | Short term memory | Reversal learning | Novelty |
| --- | --- | --- | --- | --- | --- |
| A-not-B | r_p_ = - 0.12  p = 0.46  B_01_ = 2.20 | - |  |  |  |
| Short term memory | r_p_ = 0.19  p = 0.25  B_01_ = 1.56 | r_p_ = -0.18  p = 0.28  B_01_ =1.68 | - |  |  |
| Reversal learning | **r_p_ = 0.40**  **p = 0.01**  **B_10_ = 6.18** | r_p_ = 0.20  p = 0.23  B_01_ = 1.50 | r_p_ = 0.19  p = 0.25  B_01_ = 1.56­ | - |  |
| Novelty | **r_p_ = - 0.37**  **p = 0.02**  **B_10_ = 3.66** | r_p_ = 0.06  p = 0.70  B_01_ =2.63 | r_p_ = -0.11  p = 0.50  B_01_ = 2.30 | **r_p_ = - 0.37**  **p = 0.02**  **B_10_ = 3.74** | - |
| Persistence | r_p_ = - 0.45  **p = 0.004**  B_10_ = 13.84 | r_p_ = 0.15,  p = 0.37  B_01_ = 1.97 | r_p_ = - 0.19  p = 0.25  B_01_ = 1.57 | r_p_ = - 0.15  p = 0.35  B_01_ = 1.92 | r_p_ = 0.24  p = 0.14  B_01_ =1.11 |

**Table S10. Relationships between tasks.** Bivariate correlation between tasks. The table reports the Pearson coefficient, p-value and the Bayes Factor for each comparison.

*Principal component analysis*

We next conducted a principal component analysis of the cognitive tasks, designed to cluster performance variables into sets of measures that share significant variance so we could compare these summary values across species as an additional convergent test of the ecological and social intelligence hypotheses. As mentioned previously, we used partial data from the four lemurs who did not complete the entire A-not-B task, in order to include these individuals in the PCA, given that the prior analyses validated that this inclusion did not change our main results in the A-not-B task.

We first assessed the adequacy of our correlation matrix by implementing a Bartlett's test for sphericity [24]. The test was significant (χ^2^_6_ = 13.02, p = 0.04), indicating that the correlations between measures were sufficient for principal component analysis. A principal component analysis with these variables yielded two principal unrotated components with an eigenvalue >1 (PC1 = 1.54 and PC2 = 1.19). Parallel analysis [25] confirmed retention of both components. The first principal component explained 38.5% of the variance, while the second component explained 29.7% of the variance; together both components explained ~68% of the variance (see Table S11). Although rotating the factors in PCA can often facilitates the interpretation of these factor, the information about the nature of dominant components can be lost using this approach because the total variance is redistributed amongst the rotated components more evenly than before rotation [26]. Therefore, we reported the unrotated loadings. Moreover, the scores from the first unrotated principal component have been commonly used as a measure of general intelligence in nonhuman animals [e.g., 27-28], so this approach provides for more beneficial comparisons across studies.

| Task | PC1 | PC2 |
| --- | --- | --- |
| Discounting | 0.637 | 0.026 |
| A-not-B | -0.074 | -0.823 |
| Short term memory | 0.463 | 0.384 |
| Reversal learning | 0.612 | -0.418 |
| Eigenvalue  % variance explained | 1.540  38.50 | 1.187  29.67 |

**Table S11. Principal components and loadings from the cognitive control tasks.** Most of the cognitive control variables loaded positively on the first principal component, which explained 38% of variance. The A-not-B task had a minimum contribution to the first principal component but loaded negatively on the second component, which explained 30% of the variance. N = 39 individuals.

In order to compare the performance of the four species, we then used linear regression models to test whether species varied on these summary scores (one set of models for dimension 1, and a second set for dimension 2). We found significant species’ differences for component 1, the one in which most of the tasks loaded positively: the sifakas’ score was significantly lower than the other species in dimension 1 [post-hoc comparisons in dimension 1: *sifakas/ruffed*: p = 0.008; *sifakas/mongoose*: p = 0.002; *sifakas/ring-tailed*; p < 0.001; see Table S12 for parameter of the model]. No other differences among species were found for this dimension [*ruffed/ring-tailed*: p = 0.62; *ruffed/mongoose:* p = 0.94; *ring-tailed/mongoose*: 0.92].

For dimension 2, which was mostly driven by performance in the A-not-B task, we found that mongoose lemurs had a marginally different score than sifakas [p = 0.062] and ring-tailed lemurs [p = 0.054], while ruffed lemurs showed a significantly different score than ring-tailed lemurs [p = 0.02]. No other differences among species were found for this dimension [*ruffed/mongoose*: p = 0.93; *ruffed/sifakas:* p = 0.20; *ring-tailed/sifakas*: 0.73].

| **Predictor** | **Estimate** | **S.E.** | **t** | ***p* value** |
| --- | --- | --- | --- | --- |
| Sex (reference: females) | 0.083 | 0.333 | 0.248 | 0.806 |
| Species Mongoose (reference: Sifakas) | 1.812 | 0.453 | 3.996 | **<0.001** |
| Species Ring-tailed (reference: Sifakas) | 2.108 | 0.433 | 4.872 | **<0.001** |
| Species Ruffed (reference: Sifakas) | 1.554 | 0.453 | 3.431 | **0.002** |

**Table S12. Species differences in first principal component (PC1).** Parameters from the model predicting dimension 1 of the principal component analysis.

| **Predictor** | **Estimate** | **S.E.** | **t** | ***p* value** |
| --- | --- | --- | --- | --- |
| Sex (reference: females) | -0.514 | 0.299 | -1.723 | 0.094 |
| Species Mongoose (reference: Sifakas) | -1.054 | 0.405 | -2.602 | **0.014** |
| Species Ring-tailed (reference: Sifakas) | 0.402 | 0.387 | 1.039 | 0.306 |
| Species Ruffed (reference: Sifakas) | -0.814 | 0.405 | -2.011 | **0.052** |

**Table S13. Species differences in second principal component (PC2).** Parameters from the model predicting dimension 2 of the principal component analysis.

**References**

1. Mittermeier *et al*. 2008 Lemur diversity in Madagascar. *Int. J. Primatol*., **29**, 1607-1656.

2. De Petrillo F, Rosati AG. 2020 Logical inferences from visual and auditory information in ruffed lemurs and sifakas. *Anim. Behav*., **164**, 193-204.

3. MacLean *et al.* 2014 The evolution of self-control. *PNAS*, **11**, E2140-E2148.

4. R Development Core Team. 2017 A language and environment for statistical computing. (Vienna, Austria).

5. Bates D. 2010 The LME4 package: linear mixed-effects models using S4 classes. <http://www.R-project.org>.

6. Bolker *et al.* 2008 Generalized linear mixed models: a practical guide for ecology and evolution. *Trends Ecol. Evol*., **24**, 127-135.

7. Lenth R, Singmann H, Love J, Buerkner P, Herve M. 2018 Package ‘emmeans’:

<https://cran.r-project.org/web/packages/emmeans/index.html>

8. Kuhn M, Jackson S, Cimentada J. 2020 Package ‘corr’: <https://cran.r-project.org/web/packages/corrr/index.html>

**﻿**9. Richard D. Morey *et al*. Computation of Bayes Factors for Common Designs. 2018 https://cran.r-project.org/web/packages/BayesFactor/BayesFactor.pdf

10. Rosati AG, DiNicola LM, Buckholtz JW. 2018 Chimpanzee cooperation is fast and independent from self-control. *Psychol. Sci.*, **29**, 1832-1845.

11. Rosati AG, Hare B. 2013 Chimpanzees and bonobos exhibit emotional responses to decision outcomes. *PLoS One*, **8**, e63058.

12. MacLean *et al.* 2014 The evolution of self-control. *PNAS*, **11**, E2140-E2148.

13. Rosati AG, Hare B. 2012 Chimpanzees and bonobos exhibit divergent spatial memory development. *Dev. Sci*., 15, 840-853.

14. Amici F, Aureli F, Call J. 2010 Monkeys and apes: are their cognitive skills really so different? *Am. J. Phys. Anthropol*., **143**, 188-197.

15. Many Primates *et al.* 2019 Establishing an infrastructure for collaboration in primate cognition research. *PLoS One*, **14**, e0223675.

16. Wobber V, Wrangham R, Hare B. 2010 Bonobos exhibit delayed development of social behavior and cognition relative to chimpanzees. *Curr. Biol*., **20**, 226-230.

17. Deaner RO, Van Schaik CP, Johnson V. 2006 Do some taxa have better domain-general cognition than others? A meta-analysis of nonhuman primate studies. *Evol. Psychol*., **4,** 147470490600400114.

18. Herrmann E, Hare B, Cissewski J, Tomasello M. 2011 A comparison of temperament in nonhuman apes and human infants. *Dev. Sci.*, **14**, 1393-1405.

19. Wobber V, Herrmann E, Hare B, Wrangham R, Tomasello M. 2014 Differences in the early cognitive development of children and great apes. *Dev. Psychobiol*., **56**, 547-573.

20. MacLean EL, Herrmann E, Suchindran S, Hare B. 2017 Individual differences in cooperative communicative skills are more similar between dogs and humans than chimpanzees. *Anim. Behav*., **126**, 41-51.

21. Rao A, Bernasconi L, Lazzaroni M, Marshall-Pescini S, Range F. 2018 Differences in persistence between dogs and wolves in an unsolvable task in the absence of humans. *PeerJ*, **6**, e5944.

22. Lo S, Andrews S. 2015 To transform or not to transform: Using generalized linear mixed models to analyse reaction time data. *Front. Psychol*., **6**, 1171.

23. Wetzels R, Wagenmakers EJ. 2012 A default Bayesian hypothesis test for correlations and partial correlations. *Psychon. Bull. Rev*., **19**, 1057-1064.

24. Budaev SV. 2010 Using principal components and factor analysis in animal behaviour research: caveats and guidelines. *Ethology*, **116**, 472-480.

25. Horn JL. 1965 A rationale and test for the number of factors in factor analysis. *Psychometrika*, **30**, 179-185.

26. Jolliffe IT, Cadima J. 2016 Principal component analysis: a review and recent developments. *Philos. Trans. Royal Soc. A*, **374**, 20150202.

27. Shaw RC, Boogert NJ, Clayton NS, Burns KC. 2015 Wild psychometrics: evidence for ‘general’cognitive performance in wild New Zealand robins, Petroica longipes. *Anim. Behav*., **109**, 101-111.

28. Plomin R, Spinath FM. 2002 Genetics and general cognitive ability (g). *Trends Cogn. Sci.,* **6**, 169-176.

**Supplemental movie captions**

**Video S1.** **Discounting task**. Lemurs made decisions between a smaller food treat available immediately, and a larger treat available after a delay; they also completed a pretest with no delays. Clip 1 shows a mongoose lemur in the number pretest, choosing between a larger piece of food (on the left) and a smaller piece (on the right). Here, the lemur correctly chooses the larger option on the left. Clip 2 shows a ring-tailed lemur facing a discounting choice where larger option was associated with a 15s delay; the ring-tailed lemur chooses the larger, delayed reward. Clip 3 shows a ruffed lemur facing a discounting choice and choosing the smaller, immediate option.

**Video S2. A-not-B task.** Lemurs had to resist searching for food in a previous hiding location (A) when the food reward was visibly moved to a new location (B) in order to obtain a treat. In Clip 1, a mongoose lemur chooses the correct location (container B). In Clip 2, a sifaka choose the incorrect location (container A).

**Video S3. Short term memory task.** Lemurs had to recall the location of a hidden treat either immediately after baiting (no delay), or after a 5s delay in which the lemur’ view is blocked by an occluder. In Clip 1, a ring-tailed lemur chooses the correct container in the no-delay condition. In Clip 2, a sifaka chooses the incorrect container in the no-delay condition. In Clip 3, a ruffed lemur chooses the correct container in the delay condition. In Clip 4, a mongoose lemur chooses the incorrect container in the delay condition.

**Video S4. Reversal learning task.** Lemurs initially learned that a food reward was hidden under one of two containers in learning trials, and then contingencies were switched in reversal trials. In Clip 1, a sifaka chooses the correct container (blue container) in their final learning trial, and then makes an error by choosing the same option in their first reversal trial. In Clip 2, a ruffed lemur chooses the incorrect container in a reversal trial, and then correctly updates on the next trial.

**Video S5. Novel object task.** Lemurs were presented with different stimuli to assess their willingness to approach the object; close proximity was demarcated on the table by blue tape. In Clip 1, a ruffed lemur spends relatively long time in proximity of a novel stationary object. In Clip 2, a ring-tailed lemur spends a short period time in proximity to the stationary object. In Clip 3, a sifaka spend a long time in proximity of a novel moving object. In Clip 4, a mongoose lemur spends a short period of time near the moving object.

**Video S6. Persistence task.** Lemurs first experienced retrieving a treat from a container, and then were presented with an ‘unsolvable’ version to measure their motivation to access it**.** In Clip 1, a ring-tailed lemur retrieves the food in a solvable trial, where box’s lid is unsealed. In Clip 2, a mongoose lemur spent a short time manipulating the ‘unsolvable’ trial where the lid was sealed. In Clip 3, a sifaka spends a long time attempting to open the box.
